# Measurement of the triple-differential dijet cross section in proton-proton collisions at $\sqrt{s}=8\,\text {TeV}$ and constraints on parton distribution functions

**DOI:** 10.1140/epjc/s10052-017-5286-7

**Published:** 2017-11-07

**Authors:** A. M. Sirunyan, A. Tumasyan, W. Adam, E. Asilar, T. Bergauer, J. Brandstetter, E. Brondolin, M. Dragicevic, J. Erö, M. Flechl, M. Friedl, R. Frühwirth, V. M. Ghete, C. Hartl, N. Hörmann, J. Hrubec, M. Jeitler, A. König, I. Krätschmer, D. Liko, T. Matsushita, I. Mikulec, D. Rabady, N. Rad, B. Rahbaran, H. Rohringer, J. Schieck, J. Strauss, W. Waltenberger, C.-E. Wulz, O. Dvornikov, V. Makarenko, V. Mossolov, J. Suarez Gonzalez, V. Zykunov, N. Shumeiko, S. Alderweireldt, E. A. De Wolf, X. Janssen, J. Lauwers, M. Van De Klundert, H. Van Haevermaet, P. Van Mechelen, N. Van Remortel, A. Van Spilbeeck, S. Abu Zeid, F. Blekman, J. D’Hondt, N. Daci, I. De Bruyn, K. Deroover, S. Lowette, S. Moortgat, L. Moreels, A. Olbrechts, Q. Python, K. Skovpen, S. Tavernier, W. Van Doninck, P. Van Mulders, I. Van Parijs, H. Brun, B. Clerbaux, G. De Lentdecker, H. Delannoy, G. Fasanella, L. Favart, R. Goldouzian, A. Grebenyuk, G. Karapostoli, T. Lenzi, A. Léonard, J. Luetic, T. Maerschalk, A. Marinov, A. Randle-conde, T. Seva, C. Vander Velde, P. Vanlaer, D. Vannerom, R. Yonamine, F. Zenoni, F. Zhang, T. Cornelis, D. Dobur, A. Fagot, M. Gul, I. Khvastunov, D. Poyraz, S. Salva, R. Schöfbeck, M. Tytgat, W. Van Driessche, N. Zaganidis, H. Bakhshiansohi, O. Bondu, S. Brochet, G. Bruno, A. Caudron, S. De Visscher, C. Delaere, M. Delcourt, B. Francois, A. Giammanco, A. Jafari, M. Komm, G. Krintiras, V. Lemaitre, A. Magitteri, A. Mertens, M. Musich, K. Piotrzkowski, L. Quertenmont, M. Selvaggi, M. Vidal Marono, S. Wertz, N. Beliy, W. L. Aldá Júnior, F. L. Alves, G. A. Alves, L. Brito, C. Hensel, A. Moraes, M. E. Pol, P. Rebello Teles, E. Belchior Batista Das Chagas, W. Carvalho, J. Chinellato, A. Custódio, E. M. Da Costa, G. G. Da Silveira, D. De Jesus Damiao, C. De Oliveira Martins, S. Fonseca De Souza, L. M. Huertas Guativa, H. Malbouisson, D. Matos Figueiredo, C. Mora Herrera, L. Mundim, H. Nogima, W. L. Prado Da Silva, A. Santoro, A. Sznajder, E. J. Tonelli Manganote, F. Torres Da Silva De Araujo, A. Vilela Pereira, S. Ahuja, C. A. Bernardes, S. Dogra, T. R. Fernandez Perez Tomei, E. M. Gregores, P. G. Mercadante, C. S. Moon, S. F. Novaes, Sandra S. Padula, D. Romero Abad, J. C. Ruiz Vargas, A. Aleksandrov, R. Hadjiiska, P. Iaydjiev, M. Rodozov, S. Stoykova, G. Sultanov, M. Vutova, A. Dimitrov, I. Glushkov, L. Litov, B. Pavlov, P. Petkov, W. Fang, M. Ahmad, J. G. Bian, G. M. Chen, H. S. Chen, M. Chen, Y. Chen, T. Cheng, C. H. Jiang, D. Leggat, Z. Liu, F. Romeo, M. Ruan, S. M. Shaheen, A. Spiezia, J. Tao, C. Wang, Z. Wang, E. Yazgan, H. Zhang, J. Zhao, Y. Ban, G. Chen, Q. Li, S. Liu, Y. Mao, S. J. Qian, D. Wang, Z. Xu, C. Avila, A. Cabrera, L. F. Chaparro Sierra, C. Florez, J. P. Gomez, C. F. González Hernández, J. D. Ruiz Alvarez, J. C. Sanabria, N. Godinovic, D. Lelas, I. Puljak, P. M. Ribeiro Cipriano, T. Sculac, Z. Antunovic, M. Kovac, V. Brigljevic, D. Ferencek, K. Kadija, B. Mesic, T. Susa, M. W. Ather, A. Attikis, G. Mavromanolakis, J. Mousa, C. Nicolaou, F. Ptochos, P. A. Razis, H. Rykaczewski, M. Finger, M. Finger, E. Carrera Jarrin, A. A. Abdelalim, Y. Mohammed, E. Salama, M. Kadastik, L. Perrini, M. Raidal, A. Tiko, C. Veelken, P. Eerola, J. Pekkanen, M. Voutilainen, J. Härkönen, T. Järvinen, V. Karimäki, R. Kinnunen, T. Lampén, K. Lassila-Perini, S. Lehti, T. Lindén, P. Luukka, J. Tuominiemi, E. Tuovinen, L. Wendland, J. Talvitie, T. Tuuva, M. Besancon, F. Couderc, M. Dejardin, D. Denegri, B. Fabbro, J. L. Faure, C. Favaro, F. Ferri, S. Ganjour, S. Ghosh, A. Givernaud, P. Gras, G. Hamel de Monchenault, P. Jarry, I. Kucher, E. Locci, M. Machet, J. Malcles, J. Rander, A. Rosowsky, M. Titov, A. Abdulsalam, I. Antropov, S. Baffioni, F. Beaudette, P. Busson, L. Cadamuro, E. Chapon, C. Charlot, O. Davignon, R. Granier de Cassagnac, M. Jo, S. Lisniak, P. Miné, M. Nguyen, C. Ochando, G. Ortona, P. Paganini, P. Pigard, S. Regnard, R. Salerno, Y. Sirois, A. G. Stahl Leiton, T. Strebler, Y. Yilmaz, A. Zabi, A. Zghiche, J.-L. Agram, J. Andrea, D. Bloch, J.-M. Brom, M. Buttignol, E. C. Chabert, N. Chanon, C. Collard, E. Conte, X. Coubez, J.-C. Fontaine, D. Gelé, U. Goerlach, A.-C. Le Bihan, P. Van Hove, S. Gadrat, S. Beauceron, C. Bernet, G. Boudoul, C. A. Carrillo Montoya, R. Chierici, D. Contardo, B. Courbon, P. Depasse, H. El Mamouni, J. Fay, L. Finco, S. Gascon, M. Gouzevitch, G. Grenier, B. Ille, F. Lagarde, I. B. Laktineh, M. Lethuillier, L. Mirabito, A. L. Pequegnot, S. Perries, A. Popov, V. Sordini, M. Vander Donckt, P. Verdier, S. Viret, T. Toriashvili, Z. Tsamalaidze, C. Autermann, S. Beranek, L. Feld, M. K. Kiesel, K. Klein, M. Lipinski, M. Preuten, C. Schomakers, J. Schulz, T. Verlage, A. Albert, M. Brodski, E. Dietz-Laursonn, D. Duchardt, M. Endres, M. Erdmann, S. Erdweg, T. Esch, R. Fischer, A. Güth, M. Hamer, T. Hebbeker, C. Heidemann, K. Hoepfner, S. Knutzen, M. Merschmeyer, A. Meyer, P. Millet, S. Mukherjee, M. Olschewski, K. Padeken, T. Pook, M. Radziej, H. Reithler, M. Rieger, F. Scheuch, L. Sonnenschein, D. Teyssier, S. Thüer, V. Cherepanov, G. Flügge, B. Kargoll, T. Kress, A. Künsken, J. Lingemann, T. Müller, A. Nehrkorn, A. Nowack, C. Pistone, O. Pooth, A. Stahl, M. Aldaya Martin, T. Arndt, C. Asawatangtrakuldee, K. Beernaert, O. Behnke, U. Behrens, A. A. Bin Anuar, K. Borras, A. Campbell, P. Connor, C. Contreras-Campana, F. Costanza, C. Diez Pardos, G. Dolinska, G. Eckerlin, D. Eckstein, T. Eichhorn, E. Eren, E. Gallo, J. Garay Garcia, A. Geiser, A. Gizhko, J. M. Grados Luyando, A. Grohsjean, P. Gunnellini, A. Harb, J. Hauk, M. Hempel, H. Jung, A. Kalogeropoulos, O. Karacheban, M. Kasemann, J. Keaveney, C. Kleinwort, I. Korol, D. Krücker, W. Lange, A. Lelek, T. Lenz, J. Leonard, K. Lipka, A. Lobanov, W. Lohmann, R. Mankel, I.-A. Melzer-Pellmann, A. B. Meyer, G. Mittag, J. Mnich, A. Mussgiller, D. Pitzl, R. Placakyte, A. Raspereza, B. Roland, M. Ö. Sahin, P. Saxena, T. Schoerner-Sadenius, S. Spannagel, N. Stefaniuk, G. P. Van Onsem, R. Walsh, C. Wissing, V. Blobel, M. Centis Vignali, A. R. Draeger, T. Dreyer, E. Garutti, D. Gonzalez, J. Haller, M. Hoffmann, A. Junkes, R. Klanner, R. Kogler, N. Kovalchuk, S. Kurz, T. Lapsien, I. Marchesini, D. Marconi, M. Meyer, M. Niedziela, D. Nowatschin, F. Pantaleo, T. Peiffer, A. Perieanu, C. Scharf, P. Schleper, A. Schmidt, S. Schumann, J. Schwandt, J. Sonneveld, H. Stadie, G. Steinbrück, F. M. Stober, M. Stöver, H. Tholen, D. Troendle, E. Usai, L. Vanelderen, A. Vanhoefer, B. Vormwald, M. Akbiyik, C. Barth, S. Baur, C. Baus, J. Berger, E. Butz, R. Caspart, T. Chwalek, F. Colombo, W. De Boer, A. Dierlamm, S. Fink, B. Freund, R. Friese, M. Giffels, A. Gilbert, P. Goldenzweig, D. Haitz, F. Hartmann, S. M. Heindl, U. Husemann, F. Kassel, I. Katkov, S. Kudella, H. Mildner, M. U. Mozer, Th. Müller, M. Plagge, G. Quast, K. Rabbertz, S. Röcker, F. Roscher, M. Schröder, I. Shvetsov, G. Sieber, H. J. Simonis, R. Ulrich, S. Wayand, M. Weber, T. Weiler, S. Williamson, C. Wöhrmann, R. Wolf, G. Anagnostou, G. Daskalakis, T. Geralis, V. A. Giakoumopoulou, A. Kyriakis, D. Loukas, I. Topsis-Giotis, S. Kesisoglou, A. Panagiotou, N. Saoulidou, E. Tziaferi, K. Kousouris, I. Evangelou, G. Flouris, C. Foudas, P. Kokkas, N. Loukas, N. Manthos, I. Papadopoulos, E. Paradas, N. Filipovic, G. Pasztor, G. Bencze, C. Hajdu, D. Horvath, F. Sikler, V. Veszpremi, G. Vesztergombi, A. J. Zsigmond, N. Beni, S. Czellar, J. Karancsi, A. Makovec, J. Molnar, Z. Szillasi, M. Bartók, P. Raics, Z. L. Trocsanyi, B. Ujvari, S. Choudhury, J. R. Komaragiri, S. Bahinipati, S. Bhowmik, P. Mal, K. Mandal, A. Nayak, D. K. Sahoo, N. Sahoo, S. K. Swain, S. Bansal, S. B. Beri, V. Bhatnagar, R. Chawla, U. Bhawandeep, A. K. Kalsi, A. Kaur, M. Kaur, R. Kumar, P. Kumari, A. Mehta, M. Mittal, J. B. Singh, G. Walia, Ashok Kumar, A. Bhardwaj, B. C. Choudhary, R. B. Garg, S. Keshri, A. Kumar, S. Malhotra, M. Naimuddin, K. Ranjan, R. Sharma, V. Sharma, R. Bhattacharya, S. Bhattacharya, K. Chatterjee, S. Dey, S. Dutt, S. Dutta, S. Ghosh, N. Majumdar, A. Modak, K. Mondal, S. Mukhopadhyay, S. Nandan, A. Purohit, A. Roy, D. Roy, S. Roy Chowdhury, S. Sarkar, M. Sharan, S. Thakur, P. K. Behera, R. Chudasama, D. Dutta, V. Jha, V. Kumar, A. K. Mohanty, P. K. Netrakanti, L. M. Pant, P. Shukla, A. Topkar, T. Aziz, S. Dugad, G. Kole, B. Mahakud, S. Mitra, G. B. Mohanty, B. Parida, N. Sur, B. Sutar, S. Banerjee, R. K. Dewanjee, S. Ganguly, M. Guchait, Sa. Jain, S. Kumar, M. Maity, G. Majumder, K. Mazumdar, T. Sarkar, N. Wickramage, S. Chauhan, S. Dube, V. Hegde, A. Kapoor, K. Kothekar, S. Pandey, A. Rane, S. Sharma, S. Chenarani, E. Eskandari Tadavani, S. M. Etesami, M. Khakzad, M. Mohammadi Najafabadi, M. Naseri, S. Paktinat Mehdiabadi, F. Rezaei Hosseinabadi, B. Safarzadeh, M. Zeinali, M. Felcini, M. Grunewald, M. Abbrescia, C. Calabria, C. Caputo, A. Colaleo, D. Creanza, L. Cristella, N. De Filippis, M. De Palma, L. Fiore, G. Iaselli, G. Maggi, M. Maggi, G. Miniello, S. My, S. Nuzzo, A. Pompili, G. Pugliese, R. Radogna, A. Ranieri, G. Selvaggi, A. Sharma, L. Silvestris, R. Venditti, P. Verwilligen, G. Abbiendi, C. Battilana, D. Bonacorsi, S. Braibant-Giacomelli, L. Brigliadori, R. Campanini, P. Capiluppi, A. Castro, F. R. Cavallo, S. S. Chhibra, G. Codispoti, M. Cuffiani, G. M. Dallavalle, F. Fabbri, A. Fanfani, D. Fasanella, P. Giacomelli, C. Grandi, L. Guiducci, S. Marcellini, G. Masetti, A. Montanari, F. L. Navarria, A. Perrotta, A. M. Rossi, T. Rovelli, G. P. Siroli, N. Tosi, S. Albergo, S. Costa, A. Di Mattia, F. Giordano, R. Potenza, A. Tricomi, C. Tuve, G. Barbagli, V. Ciulli, C. Civinini, R. D’Alessandro, E. Focardi, P. Lenzi, M. Meschini, S. Paoletti, L. Russo, G. Sguazzoni, D. Strom, L. Viliani, L. Benussi, S. Bianco, F. Fabbri, D. Piccolo, F. Primavera, V. Calvelli, F. Ferro, M. R. Monge, E. Robutti, S. Tosi, L. Brianza, F. Brivio, V. Ciriolo, M. E. Dinardo, S. Fiorendi, S. Gennai, A. Ghezzi, P. Govoni, M. Malberti, S. Malvezzi, R. A. Manzoni, D. Menasce, L. Moroni, M. Paganoni, D. Pedrini, S. Pigazzini, S. Ragazzi, T. Tabarelli de Fatis, S. Buontempo, N. Cavallo, G. De Nardo, S. Di Guida, F. Fabozzi, F. Fienga, A. O. M. Iorio, L. Lista, S. Meola, P. Paolucci, C. Sciacca, F. Thyssen, P. Azzi, N. Bacchetta, L. Benato, D. Bisello, A. Boletti, R. Carlin, A. Carvalho Antunes De Oliveira, P. Checchia, M. Dall’Osso, P. De Castro Manzano, T. Dorigo, U. Dosselli, F. Gasparini, U. Gasparini, A. Gozzelino, S. Lacaprara, M. Margoni, A. T. Meneguzzo, J. Pazzini, N. Pozzobon, P. Ronchese, F. Simonetto, E. Torassa, M. Zanetti, P. Zotto, G. Zumerle, A. Braghieri, F. Fallavollita, A. Magnani, P. Montagna, S. P. Ratti, V. Re, M. Ressegotti, C. Riccardi, P. Salvini, I. Vai, P. Vitulo, L. Alunni Solestizi, G. M. Bilei, D. Ciangottini, L. Fanò, P. Lariccia, R. Leonardi, G. Mantovani, V. Mariani, M. Menichelli, A. Saha, A. Santocchia, K. Androsov, P. Azzurri, G. Bagliesi, J. Bernardini, T. Boccali, R. Castaldi, M. A. Ciocci, R. Dell’Orso, G. Fedi, A. Giassi, M. T. Grippo, F. Ligabue, T. Lomtadze, L. Martini, A. Messineo, F. Palla, A. Rizzi, A. Savoy-Navarro, P. Spagnolo, R. Tenchini, G. Tonelli, A. Venturi, P. G. Verdini, L. Barone, F. Cavallari, M. Cipriani, D. Del Re, M. Diemoz, S. Gelli, E. Longo, F. Margaroli, B. Marzocchi, P. Meridiani, G. Organtini, R. Paramatti, F. Preiato, S. Rahatlou, C. Rovelli, F. Santanastasio, N. Amapane, R. Arcidiacono, S. Argiro, M. Arneodo, N. Bartosik, R. Bellan, C. Biino, N. Cartiglia, F. Cenna, M. Costa, R. Covarelli, A. Degano, N. Demaria, B. Kiani, C. Mariotti, S. Maselli, E. Migliore, V. Monaco, E. Monteil, M. Monteno, M. M. Obertino, L. Pacher, N. Pastrone, M. Pelliccioni, G. L. Pinna Angioni, F. Ravera, A. Romero, M. Ruspa, R. Sacchi, K. Shchelina, V. Sola, A. Solano, A. Staiano, P. Traczyk, S. Belforte, M. Casarsa, F. Cossutti, G. Della Ricca, A. Zanetti, D. H. Kim, G. N. Kim, M. S. Kim, J. Lee, S. Lee, S. W. Lee, Y. D. Oh, S. Sekmen, D. C. Son, Y. C. Yang, A. Lee, H. Kim, J. A. Brochero Cifuentes, T. J. Kim, S. Cho, S. Choi, Y. Go, D. Gyun, S. Ha, B. Hong, Y. Jo, Y. Kim, K. Lee, K. S. Lee, S. Lee, J. Lim, S. K. Park, Y. Roh, J. Almond, J. Kim, H. Lee, S. B. Oh, B. C. Radburn-Smith, S. h. Seo, U. K. Yang, H. D. Yoo, G. B. Yu, M. Choi, H. Kim, J. H. Kim, J. S. H. Lee, I. C. Park, G. Ryu, M. S. Ryu, Y. Choi, J. Goh, C. Hwang, J. Lee, I. Yu, V. Dudenas, A. Juodagalvis, J. Vaitkus, I. Ahmed, Z. A. Ibrahim, M. A. B. Md Ali, F. Mohamad Idris, W. A. T. Wan Abdullah, M. N. Yusli, Z. Zolkapli, H. Castilla-Valdez, E. De La Cruz-Burelo, I. Heredia-De La Cruz, R. Lopez-Fernandez, R. Magaña Villalba, J. Mejia Guisao, A. Sanchez-Hernandez, S. Carrillo Moreno, C. Oropeza Barrera, F. Vazquez Valencia, S. Carpinteyro, I. Pedraza, H. A. Salazar Ibarguen, C. Uribe Estrada, A. Morelos Pineda, D. Krofcheck, P. H. Butler, A. Ahmad, M. Ahmad, Q. Hassan, H. R. Hoorani, W. A. Khan, A. Saddique, M. A. Shah, M. Shoaib, M. Waqas, H. Bialkowska, M. Bluj, B. Boimska, T. Frueboes, M. Górski, M. Kazana, K. Nawrocki, K. Romanowska-Rybinska, M. Szleper, P. Zalewski, K. Bunkowski, A. Byszuk, K. Doroba, A. Kalinowski, M. Konecki, J. Krolikowski, M. Misiura, M. Olszewski, A. Pyskir, M. Walczak, P. Bargassa, C. Beirão Da Cruz E. Silva, B. Calpas, A. Di Francesco, P. Faccioli, M. Gallinaro, J. Hollar, N. Leonardo, L. Lloret Iglesias, M. V. Nemallapudi, J. Seixas, O. Toldaiev, D. Vadruccio, J. Varela, S. Afanasiev, P. Bunin, M. Gavrilenko, I. Golutvin, I. Gorbunov, A. Kamenev, V. Karjavin, A. Lanev, A. Malakhov, V. Matveev, V. Palichik, V. Perelygin, S. Shmatov, S. Shulha, N. Skatchkov, V. Smirnov, N. Voytishin, A. Zarubin, L. Chtchipounov, V. Golovtsov, Y. Ivanov, V. Kim, E. Kuznetsova, V. Murzin, V. Oreshkin, V. Sulimov, A. Vorobyev, Yu. Andreev, A. Dermenev, S. Gninenko, N. Golubev, A. Karneyeu, M. Kirsanov, N. Krasnikov, A. Pashenkov, D. Tlisov, A. Toropin, V. Epshteyn, V. Gavrilov, N. Lychkovskaya, V. Popov, I. Pozdnyakov, G. Safronov, A. Spiridonov, M. Toms, E. Vlasov, A. Zhokin, T. Aushev, A. Bylinkin, R. Chistov, S. Polikarpov, E. Zhemchugov, V. Andreev, M. Azarkin, I. Dremin, M. Kirakosyan, A. Leonidov, A. Terkulov, A. Baskakov, A. Belyaev, E. Boos, M. Dubinin, L. Dudko, A. Ershov, A. Gribushin, V. Klyukhin, O. Kodolova, I. Lokhtin, I. Miagkov, S. Obraztsov, S. Petrushanko, V. Savrin, A. Snigirev, V. Blinov, Y. Skovpen, D. Shtol, I. Azhgirey, I. Bayshev, S. Bitioukov, D. Elumakhov, V. Kachanov, A. Kalinin, D. Konstantinov, V. Krychkine, V. Petrov, R. Ryutin, A. Sobol, S. Troshin, N. Tyurin, A. Uzunian, A. Volkov, P. Adzic, P. Cirkovic, D. Devetak, M. Dordevic, J. Milosevic, V. Rekovic, J. Alcaraz Maestre, M. Barrio Luna, E. Calvo, M. Cerrada, M. Chamizo Llatas, N. Colino, B. De La Cruz, A. Delgado Peris, A. Escalante Del Valle, C. Fernandez Bedoya, J. P. Fernández Ramos, J. Flix, M. C. Fouz, P. Garcia-Abia, O. Gonzalez Lopez, S. Goy Lopez, J. M. Hernandez, M. I. Josa, E. Navarro De Martino, A. Pérez-Calero Yzquierdo, J. Puerta Pelayo, A. Quintario Olmeda, I. Redondo, L. Romero, M. S. Soares, J. F. de Trocóniz, M. Missiroli, D. Moran, J. Cuevas, C. Erice, J. Fernandez Menendez, I. Gonzalez Caballero, J. R. González Fernández, E. Palencia Cortezon, S. Sanchez Cruz, I. Suárez Andrés, P. Vischia, J. M. Vizan Garcia, I. J. Cabrillo, A. Calderon, E. Curras, M. Fernandez, J. Garcia-Ferrero, G. Gomez, A. Lopez Virto, J. Marco, C. Martinez Rivero, F. Matorras, J. Piedra Gomez, T. Rodrigo, A. Ruiz-Jimeno, L. Scodellaro, N. Trevisani, I. Vila, R. Vilar Cortabitarte, D. Abbaneo, E. Auffray, G. Auzinger, P. Baillon, A. H. Ball, D. Barney, P. Bloch, A. Bocci, C. Botta, T. Camporesi, R. Castello, M. Cepeda, G. Cerminara, Y. Chen, A. Cimmino, D. d’Enterria, A. Dabrowski, V. Daponte, A. David, M. De Gruttola, A. De Roeck, E. Di Marco, M. Dobson, B. Dorney, T. du Pree, D. Duggan, M. Dünser, N. Dupont, A. Elliott-Peisert, P. Everaerts, S. Fartoukh, G. Franzoni, J. Fulcher, W. Funk, D. Gigi, K. Gill, M. Girone, F. Glege, D. Gulhan, S. Gundacker, M. Guthoff, P. Harris, J. Hegeman, V. Innocente, P. Janot, J. Kieseler, H. Kirschenmann, V. Knünz, A. Kornmayer, M. J. Kortelainen, M. Krammer, C. Lange, P. Lecoq, C. Lourenço, M. T. Lucchini, L. Malgeri, M. Mannelli, A. Martelli, F. Meijers, J. A. Merlin, S. Mersi, E. Meschi, P. Milenovic, F. Moortgat, S. Morovic, M. Mulders, H. Neugebauer, S. Orfanelli, L. Orsini, L. Pape, E. Perez, M. Peruzzi, A. Petrilli, G. Petrucciani, A. Pfeiffer, M. Pierini, A. Racz, T. Reis, G. Rolandi, M. Rovere, H. Sakulin, J. B. Sauvan, C. Schäfer, C. Schwick, M. Seidel, A. Sharma, P. Silva, P. Sphicas, J. Steggemann, M. Stoye, Y. Takahashi, M. Tosi, D. Treille, A. Triossi, A. Tsirou, V. Veckalns, G. I. Veres, M. Verweij, N. Wardle, H. K. Wöhri, A. Zagozdzinska, W. D. Zeuner, W. Bertl, K. Deiters, W. Erdmann, R. Horisberger, Q. Ingram, H. C. Kaestli, D. Kotlinski, U. Langenegger, T. Rohe, S. A. Wiederkehr, F. Bachmair, L. Bäni, L. Bianchini, B. Casal, G. Dissertori, M. Dittmar, M. Donegà, C. Grab, C. Heidegger, D. Hits, J. Hoss, G. Kasieczka, W. Lustermann, B. Mangano, M. Marionneau, P. Martinez Ruiz del Arbol, M. Masciovecchio, M. T. Meinhard, D. Meister, F. Micheli, P. Musella, F. Nessi-Tedaldi, F. Pandolfi, J. Pata, F. Pauss, G. Perrin, L. Perrozzi, M. Quittnat, M. Rossini, M. Schönenberger, A. Starodumov, V. R. Tavolaro, K. Theofilatos, R. Wallny, T. K. Aarrestad, C. Amsler, L. Caminada, M. F. Canelli, A. De Cosa, S. Donato, C. Galloni, A. Hinzmann, T. Hreus, B. Kilminster, J. Ngadiuba, D. Pinna, G. Rauco, P. Robmann, D. Salerno, C. Seitz, Y. Yang, A. Zucchetta, V. Candelise, T. H. Doan, Sh. Jain, R. Khurana, M. Konyushikhin, C. M. Kuo, W. Lin, A. Pozdnyakov, S. S. Yu, Arun Kumar, P. Chang, Y. H. Chang, Y. Chao, K. F. Chen, P. H. Chen, F. Fiori, W.-S. Hou, Y. Hsiung, Y. F. Liu, R.-S. Lu, M. Miñano Moya, E. Paganis, A. Psallidas, J. f. Tsai, B. Asavapibhop, G. Singh, N. Srimanobhas, N. Suwonjandee, A. Adiguzel, M. N. Bakirci, F. Boran, S. Cerci, S. Damarseckin, Z. S. Demiroglu, C. Dozen, I. Dumanoglu, S. Girgis, G. Gokbulut, Y. Guler, I. Hos, E. E. Kangal, O. Kara, A. Kayis Topaksu, U. Kiminsu, M. Oglakci, G. Onengut, K. Ozdemir, B. Tali, S. Turkcapar, I. S. Zorbakir, C. Zorbilmez, B. Bilin, S. Bilmis, B. Isildak, G. Karapinar, M. Yalvac, M. Zeyrek, E. Gülmez, M. Kaya, O. Kaya, E. A. Yetkin, T. Yetkin, A. Cakir, K. Cankocak, S. Sen, B. Grynyov, L. Levchuk, P. Sorokin, R. Aggleton, F. Ball, L. Beck, J. J. Brooke, D. Burns, E. Clement, D. Cussans, H. Flacher, J. Goldstein, M. Grimes, G. P. Heath, H. F. Heath, J. Jacob, L. Kreczko, C. Lucas, D. M. Newbold, S. Paramesvaran, A. Poll, T. Sakuma, S. Seif El Nasr-storey, D. Smith, V. J. Smith, K. W. Bell, A. Belyaev, C. Brew, R. M. Brown, L. Calligaris, D. Cieri, D. J. A. Cockerill, J. A. Coughlan, K. Harder, S. Harper, E. Olaiya, D. Petyt, C. H. Shepherd-Themistocleous, A. Thea, I. R. Tomalin, T. Williams, M. Baber, R. Bainbridge, O. Buchmuller, A. Bundock, S. Casasso, M. Citron, D. Colling, L. Corpe, P. Dauncey, G. Davies, A. De Wit, M. Della Negra, R. Di Maria, P. Dunne, A. Elwood, D. Futyan, Y. Haddad, G. Hall, G. Iles, T. James, R. Lane, C. Laner, L. Lyons, A.-M. Magnan, S. Malik, L. Mastrolorenzo, J. Nash, A. Nikitenko, J. Pela, B. Penning, M. Pesaresi, D. M. Raymond, A. Richards, A. Rose, E. Scott, C. Seez, S. Summers, A. Tapper, K. Uchida, M. Vazquez Acosta, T. Virdee, J. Wright, S. C. Zenz, J. E. Cole, P. R. Hobson, A. Khan, P. Kyberd, I. D. Reid, P. Symonds, L. Teodorescu, M. Turner, A. Borzou, K. Call, J. Dittmann, K. Hatakeyama, H. Liu, N. Pastika, R. Bartek, A. Dominguez, A. Buccilli, S. I. Cooper, C. Henderson, P. Rumerio, C. West, D. Arcaro, A. Avetisyan, T. Bose, D. Gastler, D. Rankin, C. Richardson, J. Rohlf, L. Sulak, D. Zou, G. Benelli, D. Cutts, A. Garabedian, J. Hakala, U. Heintz, J. M. Hogan, O. Jesus, K. H. M. Kwok, E. Laird, G. Landsberg, Z. Mao, M. Narain, S. Piperov, S. Sagir, E. Spencer, R. Syarif, R. Breedon, D. Burns, M. Calderon De La Barca Sanchez, S. Chauhan, M. Chertok, J. Conway, R. Conway, P. T. Cox, R. Erbacher, C. Flores, G. Funk, M. Gardner, W. Ko, R. Lander, C. Mclean, M. Mulhearn, D. Pellett, J. Pilot, S. Shalhout, M. Shi, J. Smith, M. Squires, D. Stolp, K. Tos, M. Tripathi, M. Bachtis, C. Bravo, R. Cousins, A. Dasgupta, A. Florent, J. Hauser, M. Ignatenko, N. Mccoll, D. Saltzberg, C. Schnaible, V. Valuev, M. Weber, E. Bouvier, K. Burt, R. Clare, J. Ellison, J. W. Gary, S. M. A. Ghiasi Shirazi, G. Hanson, J. Heilman, P. Jandir, E. Kennedy, F. Lacroix, O. R. Long, M. Olmedo Negrete, M. I. Paneva, A. Shrinivas, W. Si, H. Wei, S. Wimpenny, B. R. Yates, J. G. Branson, G. B. Cerati, S. Cittolin, M. Derdzinski, R. Gerosa, A. Holzner, D. Klein, V. Krutelyov, J. Letts, I. Macneill, D. Olivito, S. Padhi, M. Pieri, M. Sani, V. Sharma, S. Simon, M. Tadel, A. Vartak, S. Wasserbaech, C. Welke, J. Wood, F. Würthwein, A. Yagil, G. Zevi Della Porta, N. Amin, R. Bhandari, J. Bradmiller-Feld, C. Campagnari, A. Dishaw, V. Dutta, M. Franco Sevilla, C. George, F. Golf, L. Gouskos, J. Gran, R. Heller, J. Incandela, S. D. Mullin, A. Ovcharova, H. Qu, J. Richman, D. Stuart, I. Suarez, J. Yoo, D. Anderson, J. Bendavid, A. Bornheim, J. Bunn, J. Duarte, J. M. Lawhorn, A. Mott, H. B. Newman, C. Pena, M. Spiropulu, J. R. Vlimant, S. Xie, R. Y. Zhu, M. B. Andrews, T. Ferguson, M. Paulini, J. Russ, M. Sun, H. Vogel, I. Vorobiev, M. Weinberg, J. P. Cumalat, W. T. Ford, F. Jensen, A. Johnson, M. Krohn, S. Leontsinis, T. Mulholland, K. Stenson, S. R. Wagner, J. Alexander, J. Chaves, J. Chu, S. Dittmer, K. Mcdermott, N. Mirman, J. R. Patterson, A. Rinkevicius, A. Ryd, L. Skinnari, L. Soffi, S. M. Tan, Z. Tao, J. Thom, J. Tucker, P. Wittich, M. Zientek, D. Winn, S. Abdullin, M. Albrow, G. Apollinari, A. Apresyan, S. Banerjee, L. A. T. Bauerdick, A. Beretvas, J. Berryhill, P. C. Bhat, G. Bolla, K. Burkett, J. N. Butler, H. W. K. Cheung, F. Chlebana, S. Cihangir, M. Cremonesi, V. D. Elvira, I. Fisk, J. Freeman, E. Gottschalk, L. Gray, D. Green, S. Grünendahl, O. Gutsche, D. Hare, R. M. Harris, S. Hasegawa, J. Hirschauer, Z. Hu, B. Jayatilaka, S. Jindariani, M. Johnson, U. Joshi, B. Klima, B. Kreis, S. Lammel, J. Linacre, D. Lincoln, R. Lipton, M. Liu, T. Liu, R. Lopes De Sá, J. Lykken, K. Maeshima, N. Magini, J. M. Marraffino, S. Maruyama, D. Mason, P. McBride, P. Merkel, S. Mrenna, S. Nahn, V. O’Dell, K. Pedro, O. Prokofyev, G. Rakness, L. Ristori, E. Sexton-Kennedy, A. Soha, W. J. Spalding, L. Spiegel, S. Stoynev, J. Strait, N. Strobbe, L. Taylor, S. Tkaczyk, N. V. Tran, L. Uplegger, E. W. Vaandering, C. Vernieri, M. Verzocchi, R. Vidal, M. Wang, H. A. Weber, A. Whitbeck, Y. Wu, D. Acosta, P. Avery, P. Bortignon, D. Bourilkov, A. Brinkerhoff, A. Carnes, M. Carver, D. Curry, S. Das, R. D. Field, I. K. Furic, J. Konigsberg, A. Korytov, J. F. Low, P. Ma, K. Matchev, H. Mei, G. Mitselmakher, D. Rank, L. Shchutska, D. Sperka, L. Thomas, J. Wang, S. Wang, J. Yelton, S. Linn, P. Markowitz, G. Martinez, J. L. Rodriguez, A. Ackert, T. Adams, A. Askew, S. Bein, S. Hagopian, V. Hagopian, K. F. Johnson, T. Kolberg, T. Perry, H. Prosper, A. Santra, R. Yohay, M. M. Baarmand, V. Bhopatkar, S. Colafranceschi, M. Hohlmann, D. Noonan, T. Roy, F. Yumiceva, M. R. Adams, L. Apanasevich, D. Berry, R. R. Betts, R. Cavanaugh, X. Chen, O. Evdokimov, C. E. Gerber, D. A. Hangal, D. J. Hofman, K. Jung, J. Kamin, I. D. Sandoval Gonzalez, H. Trauger, N. Varelas, H. Wang, Z. Wu, J. Zhang, B. Bilki, W. Clarida, K. Dilsiz, S. Durgut, R. P. Gandrajula, M. Haytmyradov, V. Khristenko, J.-P. Merlo, H. Mermerkaya, A. Mestvirishvili, A. Moeller, J. Nachtman, H. Ogul, Y. Onel, F. Ozok, A. Penzo, C. Snyder, E. Tiras, J. Wetzel, K. Yi, B. Blumenfeld, A. Cocoros, N. Eminizer, D. Fehling, L. Feng, A. V. Gritsan, P. Maksimovic, J. Roskes, U. Sarica, M. Swartz, M. Xiao, C. You, A. Al-bataineh, P. Baringer, A. Bean, S. Boren, J. Bowen, J. Castle, L. Forthomme, S. Khalil, A. Kropivnitskaya, D. Majumder, W. Mcbrayer, M. Murray, S. Sanders, R. Stringer, J. D. Tapia Takaki, Q. Wang, A. Ivanov, K. Kaadze, Y. Maravin, A. Mohammadi, L. K. Saini, N. Skhirtladze, S. Toda, F. Rebassoo, D. Wright, C. Anelli, A. Baden, O. Baron, A. Belloni, B. Calvert, S. C. Eno, C. Ferraioli, J. A. Gomez, N. J. Hadley, S. Jabeen, G. Y. Jeng, R. G. Kellogg, J. Kunkle, A. C. Mignerey, F. Ricci-Tam, Y. H. Shin, A. Skuja, M. B. Tonjes, S. C. Tonwar, D. Abercrombie, B. Allen, A. Apyan, V. Azzolini, R. Barbieri, A. Baty, R. Bi, K. Bierwagen, S. Brandt, W. Busza, I. A. Cali, M. D’Alfonso, Z. Demiragli, G. Gomez Ceballos, M. Goncharov, D. Hsu, Y. Iiyama, G. M. Innocenti, M. Klute, D. Kovalskyi, K. Krajczar, Y. S. Lai, Y.-J. Lee, A. Levin, P. D. Luckey, B. Maier, A. C. Marini, C. Mcginn, C. Mironov, S. Narayanan, X. Niu, C. Paus, C. Roland, G. Roland, J. Salfeld-Nebgen, G. S. F. Stephans, K. Tatar, D. Velicanu, J. Wang, T. W. Wang, B. Wyslouch, A. C. Benvenuti, R. M. Chatterjee, A. Evans, P. Hansen, S. Kalafut, S. C. Kao, Y. Kubota, Z. Lesko, J. Mans, S. Nourbakhsh, N. Ruckstuhl, R. Rusack, N. Tambe, J. Turkewitz, J. G. Acosta, S. Oliveros, E. Avdeeva, K. Bloom, D. R. Claes, C. Fangmeier, R. Gonzalez Suarez, R. Kamalieddin, I. Kravchenko, A. Malta Rodrigues, J. Monroy, J. E. Siado, G. R. Snow, B. Stieger, M. Alyari, J. Dolen, A. Godshalk, C. Harrington, I. Iashvili, J. Kaisen, D. Nguyen, A. Parker, S. Rappoccio, B. Roozbahani, G. Alverson, E. Barberis, A. Hortiangtham, A. Massironi, D. M. Morse, D. Nash, T. Orimoto, R. Teixeira De Lima, D. Trocino, R.-J. Wang, D. Wood, S. Bhattacharya, O. Charaf, K. A. Hahn, N. Mucia, N. Odell, B. Pollack, M. H. Schmitt, K. Sung, M. Trovato, M. Velasco, N. Dev, M. Hildreth, K. Hurtado Anampa, C. Jessop, D. J. Karmgard, N. Kellams, K. Lannon, N. Marinelli, F. Meng, C. Mueller, Y. Musienko, M. Planer, A. Reinsvold, R. Ruchti, N. Rupprecht, G. Smith, S. Taroni, M. Wayne, M. Wolf, A. Woodard, J. Alimena, L. Antonelli, B. Bylsma, L. S. Durkin, S. Flowers, B. Francis, A. Hart, C. Hill, W. Ji, B. Liu, W. Luo, D. Puigh, B. L. Winer, H. W. Wulsin, S. Cooperstein, O. Driga, P. Elmer, J. Hardenbrook, P. Hebda, D. Lange, J. Luo, D. Marlow, T. Medvedeva, K. Mei, I. Ojalvo, J. Olsen, C. Palmer, P. Piroué, D. Stickland, A. Svyatkovskiy, C. Tully, S. Malik, A. Barker, V. E. Barnes, S. Folgueras, L. Gutay, M. K. Jha, M. Jones, A. W. Jung, A. Khatiwada, D. H. Miller, N. Neumeister, J. F. Schulte, X. Shi, J. Sun, F. Wang, W. Xie, N. Parashar, J. Stupak, A. Adair, B. Akgun, Z. Chen, K. M. Ecklund, F. J. M. Geurts, M. Guilbaud, W. Li, B. Michlin, M. Northup, B. P. Padley, J. Roberts, J. Rorie, Z. Tu, J. Zabel, B. Betchart, A. Bodek, P. de Barbaro, R. Demina, Y. t. Duh, T. Ferbel, M. Galanti, A. Garcia-Bellido, J. Han, O. Hindrichs, A. Khukhunaishvili, K. H. Lo, P. Tan, M. Verzetti, A. Agapitos, J. P. Chou, Y. Gershtein, T. A. Gómez Espinosa, E. Halkiadakis, M. Heindl, E. Hughes, S. Kaplan, R. Kunnawalkam Elayavalli, S. Kyriacou, A. Lath, R. Montalvo, K. Nash, M. Osherson, H. Saka, S. Salur, S. Schnetzer, D. Sheffield, S. Somalwar, R. Stone, S. Thomas, P. Thomassen, M. Walker, A. G. Delannoy, M. Foerster, J. Heideman, G. Riley, K. Rose, S. Spanier, K. Thapa, O. Bouhali, A. Celik, M. Dalchenko, M. De Mattia, A. Delgado, S. Dildick, R. Eusebi, J. Gilmore, T. Huang, E. Juska, T. Kamon, R. Mueller, Y. Pakhotin, R. Patel, A. Perloff, L. Perniè, D. Rathjens, A. Safonov, A. Tatarinov, K. A. Ulmer, N. Akchurin, J. Damgov, F. De Guio, C. Dragoiu, P. R. Dudero, J. Faulkner, E. Gurpinar, S. Kunori, K. Lamichhane, S. W. Lee, T. Libeiro, T. Peltola, S. Undleeb, I. Volobouev, Z. Wang, S. Greene, A. Gurrola, R. Janjam, W. Johns, C. Maguire, A. Melo, H. Ni, P. Sheldon, S. Tuo, J. Velkovska, Q. Xu, M. W. Arenton, P. Barria, B. Cox, R. Hirosky, A. Ledovskoy, H. Li, C. Neu, T. Sinthuprasith, X. Sun, Y. Wang, E. Wolfe, F. Xia, C. Clarke, R. Harr, P. E. Karchin, J. Sturdy, S. Zaleski, D. A. Belknap, J. Buchanan, C. Caillol, S. Dasu, L. Dodd, S. Duric, B. Gomber, M. Grothe, M. Herndon, A. Hervé, U. Hussain, P. Klabbers, A. Lanaro, A. Levine, K. Long, R. Loveless, G. A. Pierro, G. Polese, T. Ruggles, A. Savin, N. Smith, W. H. Smith, D. Taylor, N. Woods, CMS Collaboration

**Affiliations:** 1 Yerevan Physics Institute, Yerevan, Armenia; 2 Institut für Hochenergiephysik, Vienna, Austria; 3 Institute for Nuclear Problems, Minsk, Belarus; 4 National Centre for Particle and High Energy Physics, Minsk, Belarus; 5 Universiteit Antwerpen, Antwerpen, Belgium; 6 Vrije Universiteit Brussel, Brussels, Belgium; 7 Université Libre de Bruxelles, Brussels, Belgium; 8 Ghent University, Ghent, Belgium; 9 Université Catholique de Louvain, Louvain-la-Neuve, Belgium; 10 Université de Mons, Mons, Belgium; 11 Centro Brasileiro de Pesquisas Fisicas, Rio de Janeiro, Brazil; 12 Universidade do Estado do Rio de Janeiro, Rio de Janeiro, Brazil; 13 Universidade Estadual Paulista, Universidade Federal do ABC, São Paulo, Brazil; 14 Institute for Nuclear Research and Nuclear Energy, Sofia, Bulgaria; 15 University of Sofia, Sofia, Bulgaria; 16 Beihang University, Beijing, China; 17 Institute of High Energy Physics, Beijing, China; 18 State Key Laboratory of Nuclear Physics and Technology, Peking University, Beijing, China; 19 Universidad de Los Andes, Bogotá, Colombia; 20 Faculty of Electrical Engineering, Mechanical Engineering and Naval Architecture, University of Split, Split, Croatia; 21 Faculty of Science, University of Split, Split, Croatia; 22 Institute Rudjer Boskovic, Zagreb, Croatia; 23 University of Cyprus, Nicosia, Cyprus; 24 Charles University, Prague, Czech Republic; 25 Universidad San Francisco de Quito, Quito, Ecuador; 26 Academy of Scientific Research and Technology of the Arab Republic of Egypt, Egyptian Network of High Energy Physics, Cairo, Egypt; 27 National Institute of Chemical Physics and Biophysics, Tallinn, Estonia; 28 Department of Physics, University of Helsinki, Helsinki, Finland; 29 Helsinki Institute of Physics, Helsinki, Finland; 30 Lappeenranta University of Technology, Lappeenranta, Finland; 31 IRFU, CEA, Université Paris-Saclay, Gif-sur-Yvette, France; 32 Laboratoire Leprince-Ringuet, Ecole polytechnique, CNRS/IN2P3, Université Paris-Saclay, Palaiseau, France; 33 Université de Strasbourg, CNRS, IPHC UMR 7178, 67000 Strasbourg, France; 34 Centre de Calcul de l’Institut National de Physique Nucleaire et de Physique des Particules, CNRS/IN2P3, Villeurbanne, France; 35 Institut de Physique Nucléaire de Lyon, Université de Lyon, Université Claude Bernard Lyon 1, CNRS-IN2P3, Villeurbanne, France; 36 Georgian Technical University, Tbilisi, Georgia; 37 Tbilisi State University, Tbilisi, Georgia; 38 RWTH Aachen University, I. Physikalisches Institut, Aachen, Germany; 39 RWTH Aachen University, III. Physikalisches Institut A, Aachen, Germany; 40 RWTH Aachen University, III. Physikalisches Institut B, Aachen, Germany; 41 Deutsches Elektronen-Synchrotron, Hamburg, Germany; 42 University of Hamburg, Hamburg, Germany; 43 Institut für Experimentelle Kernphysik, Karlsruhe, Germany; 44 Institute of Nuclear and Particle Physics (INPP), NCSR Demokritos, Aghia Paraskevi, Greece; 45 National and Kapodistrian University of Athens, Athens, Greece; 46 National Technical University of Athens, Athens, Greece; 47 University of Ioánnina, Ioannina, Greece; 48 MTA-ELTE Lendület CMS Particle and Nuclear Physics Group, Eötvös Loránd University, Budapest, Hungary; 49 Wigner Research Centre for Physics, Budapest, Hungary; 50 Institute of Nuclear Research ATOMKI, Debrecen, Hungary; 51 Institute of Physics, University of Debrecen, Debrecen, Hungary; 52 Indian Institute of Science (IISc), Bangalore, India; 53 National Institute of Science Education and Research, Bhubaneswar, India; 54 Panjab University, Chandigarh, India; 55 University of Delhi, Delhi, India; 56 Saha Institute of Nuclear Physics, Kolkata, India; 57 Indian Institute of Technology Madras, Madras, India; 58 Bhabha Atomic Research Centre, Mumbai, India; 59 Tata Institute of Fundamental Research-A, Mumbai, India; 60 Tata Institute of Fundamental Research-B, Mumbai, India; 61 Indian Institute of Science Education and Research (IISER), Pune, India; 62 Institute for Research in Fundamental Sciences (IPM), Tehran, Iran; 63 University College Dublin, Dublin, Ireland; 64 INFN Sezione di Bari, Università di Bari, Politecnico di Bari, Bari, Italy; 65 INFN Sezione di Bologna, Università di Bologna, Bologna, Italy; 66 INFN Sezione di Catania, Università di Catania, Catania, Italy; 67 INFN Sezione di Firenze, Università di Firenze, Florence, Italy; 68 INFN Laboratori Nazionali di Frascati, Frascati, Italy; 69 INFN Sezione di Genova, Università di Genova, Genoa, Italy; 70 INFN Sezione di Milano-Bicocca, Università di Milano-Bicocca, Milan, Italy; 71 INFN Sezione di Napoli, Università di Napoli ’Federico II’ , Napoli, Italy, Università della Basilicata, Potenza, Italy, Università G. Marconi, Rome, Italy; 72 INFN Sezione di Padova, Università di Padova, Padova, Italy, Università di Trento, Trento, Italy; 73 INFN Sezione di Pavia, Università di Pavia, Pavia, Italy; 74 INFN Sezione di Perugia, Università di Perugia, Perugia, Italy; 75 INFN Sezione di Pisa, Università di Pisa, Scuola Normale Superiore di Pisa, Pisa, Italy; 76 INFN Sezione di Roma, Sapienza Università di Roma, Rome, Italy; 77 INFN Sezione di Torino, Università di Torino, Torino, Italy, Università del Piemonte Orientale, Novara, Italy; 78 INFN Sezione di Trieste, Università di Trieste, Trieste, Italy; 79 Kyungpook National University, Daegu, Korea; 80 Chonbuk National University, Jeonju, Korea; 81 Institute for Universe and Elementary Particles, Chonnam National University, Kwangju, Korea; 82 Hanyang University, Seoul, Korea; 83 Korea University, Seoul, Korea; 84 Seoul National University, Seoul, Korea; 85 University of Seoul, Seoul, Korea; 86 Sungkyunkwan University, Suwon, Korea; 87 Vilnius University, Vilnius, Lithuania; 88 National Centre for Particle Physics, Universiti Malaya, Kuala Lumpur, Malaysia; 89 Centro de Investigacion y de Estudios Avanzados del IPN, Mexico City, Mexico; 90 Universidad Iberoamericana, Mexico City, Mexico; 91 Benemerita Universidad Autonoma de Puebla, Puebla, Mexico; 92 Universidad Autónoma de San Luis Potosí, San Luis Potosí, Mexico; 93 University of Auckland, Auckland, New Zealand; 94 University of Canterbury, Christchurch, New Zealand; 95 National Centre for Physics, Quaid-I-Azam University, Islamabad, Pakistan; 96 National Centre for Nuclear Research, Swierk, Poland; 97 Faculty of Physics, Institute of Experimental Physics, University of Warsaw, Warsaw, Poland; 98 Laboratório de Instrumentação e Física Experimental de Partículas, Lisbon, Portugal; 99 Joint Institute for Nuclear Research, Dubna, Russia; 100 Petersburg Nuclear Physics Institute, Gatchina, St. Petersburg, Russia; 101 Institute for Nuclear Research, Moscow, Russia; 102 Institute for Theoretical and Experimental Physics, Moscow, Russia; 103 Moscow Institute of Physics and Technology, Moscow, Russia; 104 National Research Nuclear University ‘Moscow Engineering Physics Institute’ (MEPhI), Moscow, Russia; 105 P.N. Lebedev Physical Institute, Moscow, Russia; 106 Skobeltsyn Institute of Nuclear Physics, Lomonosov Moscow State University, Moscow, Russia; 107 Novosibirsk State University (NSU), Novosibirsk, Russia; 108 State Research Center of Russian Federation, Institute for High Energy Physics, Protvino, Russia; 109 Faculty of Physics and Vinca Institute of Nuclear Sciences, University of Belgrade, Belgrade, Serbia; 110 Centro de Investigaciones Energéticas Medioambientales y Tecnológicas (CIEMAT), Madrid, Spain; 111 Universidad Autónoma de Madrid, Madrid, Spain; 112 Universidad de Oviedo, Oviedo, Spain; 113 Instituto de Física de Cantabria (IFCA), CSIC-Universidad de Cantabria, Santander, Spain; 114 CERN, European Organization for Nuclear Research, Geneva, Switzerland; 115 Paul Scherrer Institut, Villigen, Switzerland; 116 Institute for Particle Physics, ETH Zurich, Zurich, Switzerland; 117 Universität Zürich, Zurich, Switzerland; 118 National Central University, Chung-Li, Taiwan; 119 National Taiwan University (NTU), Taipei, Taiwan; 120 Department of Physics, Faculty of Science, Chulalongkorn University, Bangkok, Thailand; 121 Physics Department, Science and Art Faculty, Cukurova University, Adana, Turkey; 122 Physics Department, Middle East Technical University, Ankara, Turkey; 123 Bogazici University, Istanbul, Turkey; 124 Istanbul Technical University, Istanbul, Turkey; 125 Institute for Scintillation Materials of National Academy of Science of Ukraine, Kharkov, Ukraine; 126 National Scientific Center, Kharkov Institute of Physics and Technology, Kharkov, Ukraine; 127 University of Bristol, Bristol, UK; 128 Rutherford Appleton Laboratory, Didcot, UK; 129 Imperial College, London, UK; 130 Brunel University, Uxbridge, UK; 131 Baylor University, Waco, USA; 132 Catholic University of America, Washington, USA; 133 The University of Alabama, Tuscaloosa, USA; 134 Boston University, Boston, USA; 135 Brown University, Providence, USA; 136 University of California, Davis, Davis USA; 137 University of California, Los Angeles, USA; 138 University of California, Riverside, Riverside USA; 139 University of California, San Diego, La Jolla USA; 140 Department of Physics, University of California, Santa Barbara, USA; 141 California Institute of Technology, Pasadena, USA; 142 Carnegie Mellon University, Pittsburgh, USA; 143 University of Colorado Boulder, Boulder, USA; 144 Cornell University, Ithaca, USA; 145 Fairfield University, Fairfield, USA; 146 Fermi National Accelerator Laboratory, Batavia, USA; 147 University of Florida, Gainesville, USA; 148 Florida International University, Miami, USA; 149 Florida State University, Tallahassee, USA; 150 Florida Institute of Technology, Melbourne, USA; 151 University of Illinois at Chicago (UIC), Chicago, USA; 152 The University of Iowa, Iowa City, USA; 153 Johns Hopkins University, Baltimore, USA; 154 The University of Kansas, Lawrence, USA; 155 Kansas State University, Manhattan, USA; 156 Lawrence Livermore National Laboratory, Livermore, USA; 157 University of Maryland, College Park, USA; 158 Massachusetts Institute of Technology, Cambridge, USA; 159 University of Minnesota, Minneapolis, USA; 160 University of Mississippi, Oxford, USA; 161 University of Nebraska-Lincoln, Lincoln, USA; 162 State University of New York, Buffalo, USA; 163 Northeastern University, Boston, USA; 164 Northwestern University, Evanston, USA; 165 University of Notre Dame, Notre Dame, USA; 166 The Ohio State University, Columbus, USA; 167 Princeton University, Princeton, USA; 168 University of Puerto Rico, Mayaguez, USA; 169 Purdue University, West Lafayette, USA; 170 Purdue University Northwest, Hammond, USA; 171 Rice University, Houston, USA; 172 University of Rochester, Rochester, USA; 173 Rutgers, The State University of New Jersey, Piscataway, USA; 174 University of Tennessee, Knoxville, USA; 175 Texas A&M University, College Station, USA; 176 Texas Tech University, Lubbock, USA; 177 Vanderbilt University, Nashville, USA; 178 University of Virginia, Charlottesville, USA; 179 Wayne State University, Detroit, USA; 180 University of Wisconsin-Madison, Madison, WI USA; 181 CERN, Geneva, Switzerland

**Keywords:** CMS, Physics, QCD, PDF, Jets, Strong coupling constant, alpha-s

## Abstract

A measurement is presented of the triple-differential dijet cross section at a centre-of-mass energy of 8$\,\text {TeV}$ using 19.7$\,\text {fb}^\text {-1}$ of data collected with the CMS detector in proton-proton collisions at the LHC. The cross section is measured as a function of the average transverse momentum, half the rapidity separation, and the boost of the two leading jets in the event. The cross section is corrected for detector effects and compared to calculations in perturbative quantum chromodynamics at next-to-leading order accuracy, complemented with electroweak and nonperturbative corrections. New constraints on parton distribution functions are obtained and the inferred value of the strong coupling constant is $\alpha _S(M_\text {Z}) = 0.1199\,\pm {0.0015}\,(\mathrm {exp})\, _{-0.0020}^{+0.0031}\,(\mathrm {theo})$, where $M_\text {Z}$ is the mass of the Z boson.

## Introduction

The pairwise production of hadronic jets is one of the fundamental processes studied at hadron colliders. Dijet events with large transverse momenta can be described by parton-parton scattering in the context of quantum chromodynamics (QCD). Measurements of dijet cross sections can be used to thoroughly test predictions of perturbative QCD (pQCD) at high energies and to constrain parton distribution functions (PDFs). Previous measurements of dijet cross sections in proton-(anti)proton collisions have been performed as a function of dijet mass at the Sp$\bar{\text {p}}$S, ISR, and Tevatron colliders [1–6]. At the CERN LHC, dijet measurements as a function of dijet mass are reported in Refs. [7–11]. Also, dijet events have been studied triple-differentially in transverse energy and pseudorapidities $\eta _1$ and $\eta _2$ of the two leading jets [12, 13].

**Fig. 1:**
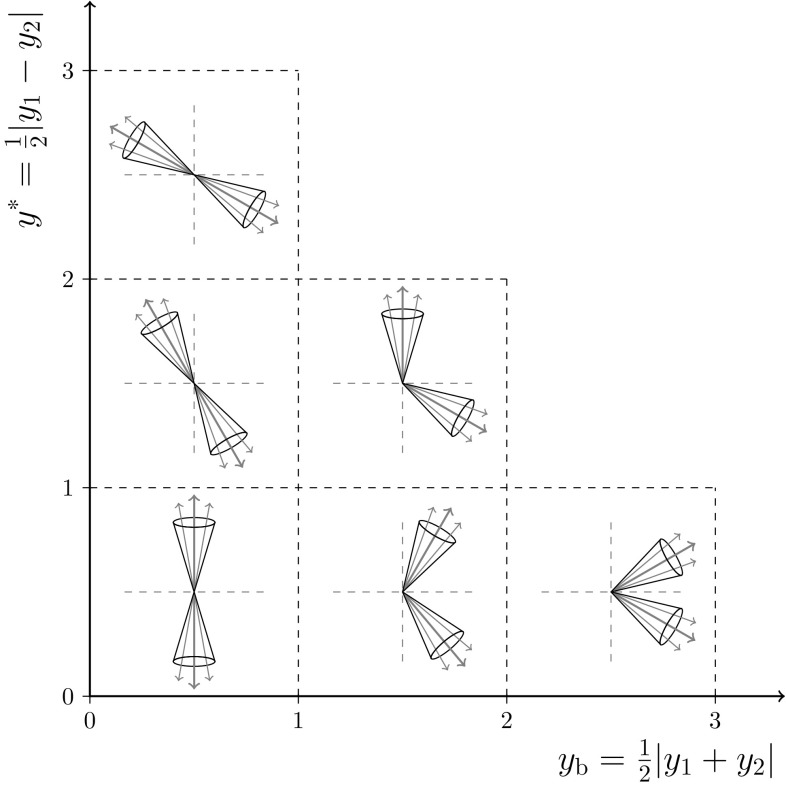
Illustration of the dijet event topologies in the $y^{*}$ and $y_{\mathrm {b}}$ kinematic plane. The dijet system can be classified as a same-side or opposite-side jet event according to the boost $y_{\mathrm {b}}$ of the two leading jets, thereby providing insight into the parton kinematics

In this paper, a measurement of the triple-differential dijet cross section is presented as a function of the average transverse momentum $p_{\mathrm {T,avg}} = (p_{\mathrm {T},1} + p_{\mathrm {T},2}) / 2$ of the two leading jets, half of their rapidity separation $y^{*} = |y_1 - y_2| / 2$, and the boost of the dijet system $y_{\mathrm {b}} = |y_1 + y_2| / 2$. The dijet event topologies are illustrated in Fig. 1.

The relation between the dijet rapidities and the parton momentum fractions $x_{1,2}$ of the incoming protons at leading order (LO) is given by $x_{1,2} = \frac{p_{\mathrm {T}}}{\sqrt{s}} ( e^{\pm y_1} + e^{\pm y_2})$, where $p_{\mathrm {T}} = p_{\mathrm {T},1} = p_{\mathrm {T},2}$. For large values of $y_{\mathrm {b}}$, the momentum fractions carried by the incoming partons must correspond to one large and one small value, while for small $y_{\mathrm {b}}$ the momentum fractions must be approximately equal. In addition, for high transverse momenta of the jets, *x* values are probed above 0.1, where the proton PDFs are less precisely known.

**Fig. 2:**
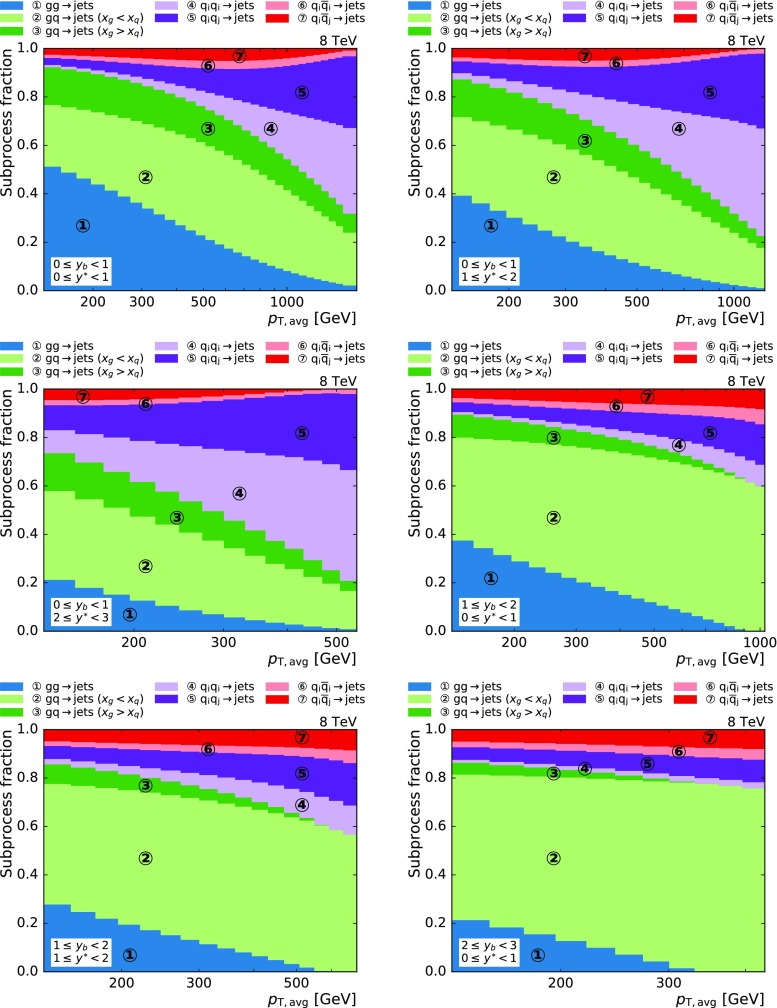
Relative contributions of all subprocesses to the total cross section at NLO as a function of $p_{\mathrm {T,avg}}$ in the various $y^{*}$ and $y_{\mathrm {b}}$ bins. The subprocess contributions are grouped into seven categories according to the type of the incoming partons. The calculations have been performed with NLOJet++. The notation implies the sum over initial-state parton flavors as well as interchanged quarks and antiquarks

The decomposition of the dijet cross section into the contributing partonic subprocesses is shown in Fig. 2 at next-to-leading order (NLO) accuracy, obtained using the NLOJet++ program version 4.1.3 [14, 15]. At small $y_{\mathrm {b}}$ and large $p_{\mathrm {T,avg}}$ a significant portion of the cross section corresponds to quark-quark (and small amounts of antiquark-antiquark) scattering with varying shares of equal- or unequal-type quarks. In contrast, for large $y_{\mathrm {b}}$ more than 80% of the cross section corresponds to partonic subprocesses with at least one gluon participating in the interaction. As a consequence, new information about the PDFs can be derived from the measurement of the triple-differential dijet cross section.

The data were collected with the CMS detector at $\sqrt{s} = 8\,\text {TeV}$ and correspond to an integrated luminosity of 19.7$\,\text {fb}^\text {-1}$. The measured cross section is corrected for detector effects and is compared to NLO calculations in pQCD, complemented with electroweak (EW) and nonperturbative (NP) corrections. Furthermore, constraints on the PDFs are studied and the strong coupling constant $\alpha _S (M_\mathrm {Z})$ is inferred.

## The CMS detector

The central feature of the CMS apparatus is a superconducting solenoid of 6$\text {\,m}$ internal diameter, providing a magnetic field of 3.8$\text {\,T}$. Within the solenoid volume are a silicon pixel and strip tracker, a lead tungstate crystal electromagnetic calorimeter (ECAL), and a brass and scintillator hadron calorimeter (HCAL), each composed of a barrel and two endcap sections. The silicon tracker measures charged particles within the pseudorapidity range $|\eta | < 2.5$. It consists of 1440 silicon pixel and 15 148 silicon strip detector modules. The ECAL consists of 75 848 lead tungstate crystals, which provide coverage in pseudorapidity $|\eta | < 1.48$ in a barrel region and $1.48< |\eta | < 3.0$ in two endcap regions. In the region $|\eta | < 1.74$, the HCAL cells have widths of 0.087 in pseudorapidity and 0.087 in azimuth ($\phi$). In the $\eta$-$\phi$ plane, and for $|\eta | < 1.48$, the HCAL cells map on to $5\times {}5$ arrays of ECAL crystals to form calorimeter towers projecting radially outwards from close to the nominal interaction point. For $|\eta | > 1.74$, the coverage of the towers increases progressively to a maximum of 0.174 in $\varDelta \eta$ and $\varDelta \phi$. Within each tower, the energy deposits in ECAL and HCAL cells are summed to define the calorimeter tower energies, subsequently used to provide the energies and directions of hadronic jets. The forward hadron (HF) calorimeter extends the pseudorapidity coverage provided by the barrel and endcap detectors and uses steel as an absorber and quartz fibers as the sensitive material. The two halves of the HF are located 11.2$\text {\,m}$ from the interaction region, one on each end, and together they provide coverage in the range $3.0< |\eta | < 5.2$. Muons are measured in gas-ionisation detectors embedded in the steel flux-return yoke outside the solenoid.

A more detailed description of the CMS detector, together with a definition of the coordinate system used and the relevant kinematic variables, can be found in Ref. [16].

## Event reconstruction and selection

Dijet events are collected using five single-jet high-level triggers [17, 18], which require at least one jet with $p_{\mathrm {T}}$ larger than 80, 140, 200, 260, and 320$\,\text {GeV}$, respectively. At trigger level the jets are reconstructed with a simplified version of the particle-flow (PF) event reconstruction described in the following paragraph. All but the highest threshold trigger were prescaled in the 2012 LHC run. The triggers are employed in mutually exclusive regions of the $p_{\mathrm {T,avg}}$ spectrum, cf. Table 1, in which their efficiency exceeds 99%.

**Table 1:** List of single-jet trigger thresholds used in the analysis

Trigger threshold [GeV]	$p_{\mathrm {T,avg}}$ range [GeV]
80	123–192
140	192–263
200	263–353
260	353–412
320	${>} 412$

The PF event algorithm reconstructs and identifies particle candidates with an optimised combination of information from the various elements of the CMS detector [19]. The energy of photons is directly obtained from the ECAL measurement, corrected for zero-suppression effects. The energy of electrons is determined from a combination of the electron momentum at the primary interaction vertex as determined by the tracker, the energy of the corresponding ECAL cluster, and the energy sum of all bremsstrahlung photons spatially compatible with originating from the electron track. The energy of muons is obtained from the curvature of the corresponding track. The energy of charged hadrons is determined from a combination of their momentum measured in the tracker and the matching ECAL and HCAL energy deposits, corrected for zero-suppression effects and for the response function of the calorimeters to hadronic showers. Finally, the energy of neutral hadrons is obtained from the corresponding corrected ECAL and HCAL energies. The leading primary vertex (PV) is chosen as the one with the highest sum of squares of all associated track transverse momenta. The remaining vertices are classified as pileup vertices, which result from additional proton-proton collisions. To reduce the background caused by such additional collisions, charged hadrons within the coverage of the tracker, $|\eta | < 2.5$ [20], that unambiguously originate from a pileup vertex are removed.

Hadronic jets are clustered from the reconstructed particles with the infrared- and collinear-safe anti-$k_{\mathrm {T}}$ algorithm [21] with a jet size parameter *R* of 0.7, which is the default for CMS jet measurements. The jet momentum is determined as the vectorial sum of all particle momenta in the jet, and is found in the simulation to be within 5–10% of the true momentum over the whole $p_{\mathrm {T}}$ range. Jet energy corrections (JEC) are derived from the simulation, and are confirmed with in situ measurements of the energy balance of dijet, photon+jet, and Z boson+jet events [22, 23]. After applying the usual jet energy corrections, a small bias in the reconstructed pseudorapidity of the jets is observed at the edge of the tracker. An additional correction removes this effect.

All events are required to have at least one PV that must be reconstructed from four or more tracks. The longitudinal and transverse distances of the PV to the nominal interaction point of CMS must satisfy $|z_\mathrm {PV}| < 24 \,\text {cm}$ and $\rho _\mathrm {PV} < 2 \,\text {cm}$, respectively. Nonphysical jets are removed by loose jet identification criteria: each jet must contain at least two PF candidates, one of which is a charged hadron, and the jet energy fraction carried by neutral hadrons and photons must be less than 99%. These criteria remove less than 1% of genuine jets.

Only events with at least two jets up to an absolute rapidity of $|y|=5.0$ are selected and the two jets leading in $p_{\mathrm {T}}$ are required to have transverse momenta greater than 50$\,\text {GeV}$ and $|y| < 3.0$. The missing transverse momentum is defined as the negative vector sum of the transverse momenta of all PF candidates in the event. Its magnitude is referred to as $p_{\mathrm {T}} ^\text {miss}$. For consistency with previous jet measurements by CMS, $p_{\mathrm {T}} ^\text {miss}$ is required to be smaller than 30% of the scalar sum of the transverse momenta of all PF candidates. For dijet events, which exhibit very little $p_{\mathrm {T}}$ imbalance, the impact is practically negligible.

## Measurement of the triple-differential dijet cross section

The triple-differential cross section for dijet production is defined as

$$\begin{aligned} \frac{\mathrm {d}^3 \sigma }{\mathrm {d}p_{\mathrm {T,avg}} \mathrm {d}y^{*} \mathrm {d}y_{\mathrm {b}}} = \frac{1}{\epsilon \mathcal {L}_{\mathrm {int}}^\mathrm {eff}} \frac{N}{\varDelta p_{\mathrm {T,avg}} \varDelta y^{*} \varDelta y_{\mathrm {b}}}, \end{aligned}$$

where *N* denotes the number of dijet events within a given bin, $\mathcal {L}_{\mathrm {int}}^{\mathrm {eff}}$ the effective integrated luminosity, and $\epsilon$ the product of trigger and event selection efficiencies, which are greater than 99% in the phase space of the measurement. Contributions from background processes, such as $\mathrm {t}\overline{\mathrm {t}}$ production, are several orders of magnitude smaller and are neglected. The bin widths are $\varDelta p_{\mathrm {T,avg}}$, $\varDelta y^{*}$, and $\varDelta y_{\mathrm {b}}$.

The cross section is unfolded to the stable-particle level (lifetime $c\tau > 1\,\text {cm}$) to correct for detector resolution effects. The iterative D’Agostini algorithm with early stopping [24–26], as implemented in the RooUnfold package [27], is employed for the unfolding. The response matrix, which relates the particle-level distribution to the measured distribution at detector level, is derived using a forward smearing technique. An NLOJet++ prediction, obtained with CT14 PDFs [28] and corrected for NP and EW effects, is approximated by a continuous function to represent the distribution at particle level. Subsequently, pseudoevents are distributed uniformly in $p_{\mathrm {T,avg}}$ and weighted according to the theoretical prediction. These weighted events are smeared using the jet $p_{\mathrm {T}}$ resolution to yield a response matrix and a prediction at detector level. By using large numbers of such pseudoevents, statistical fluctuations in the response matrix are strongly suppressed.

The jet energy (or $p_{\mathrm {T}}$) resolution (JER) is determined from the CMS detector simulation based on the Geant4 toolkit [29] and the pythia  6.4 Monte Carlo (MC) event generator [30] and is corrected for residual differences between data and simulation following Ref. [23]. The rapidity dependence of both the JER from simulation and of the residual differences have been taken into account. The Gaussian $p_{\mathrm {T}}$ resolution in the interval $|y|<1$ is about 8% at 100$\,\text {GeV}$ and improves to 5% at 1$\,\text {TeV}$. Non-Gaussian tails in the JER, exhibited for jet rapidities close to $|y|=3$, are included in a corresponding uncertainty.

The regularisation strength of the iterative unfolding procedure is defined through the number of iterations, whose optimal value is determined by performing a $\chi ^2$ test between the original measured data and the unfolded data after smearing with the response matrix. The values obtained for $\chi ^2$ per number of degrees of freedom, $n_{\mathrm {dof}}$, in these comparisons approach unity in four iterations and thereafter decrease slowly for additional iterations. The optimal number of iterations is therefore determined to be four. The procedure is in agreement with the criteria of Ref. [31]. The response matrices derived in this manner for each bin in $y^{*}$ and $y_{\mathrm {b}}$ are nearly diagonal. A cross check using the pythia 6 MC event generator as theory and the detector simulation to construct the response matrices revealed no discrepancies compared to the baseline result.

Migrations into and out of the accepted phase space in $y^{*}$ and $y_{\mathrm {b}}$ or between bins happen only at a level below 5%. The net effect of these migrations has been included in the respective response matrices and has been cross checked successfully using a 3-dimensional unfolding.

As a consequence of these migrations, small statistical correlations between neighbouring bins of the unfolded cross sections are introduced during the unfolding procedure. The statistical uncertainties after being propagated through the unfolding are smaller than 1% in the majority of the phase space, and amount up to 20% for highest $p_{\mathrm {T,avg}}$.

**Fig. 3:**
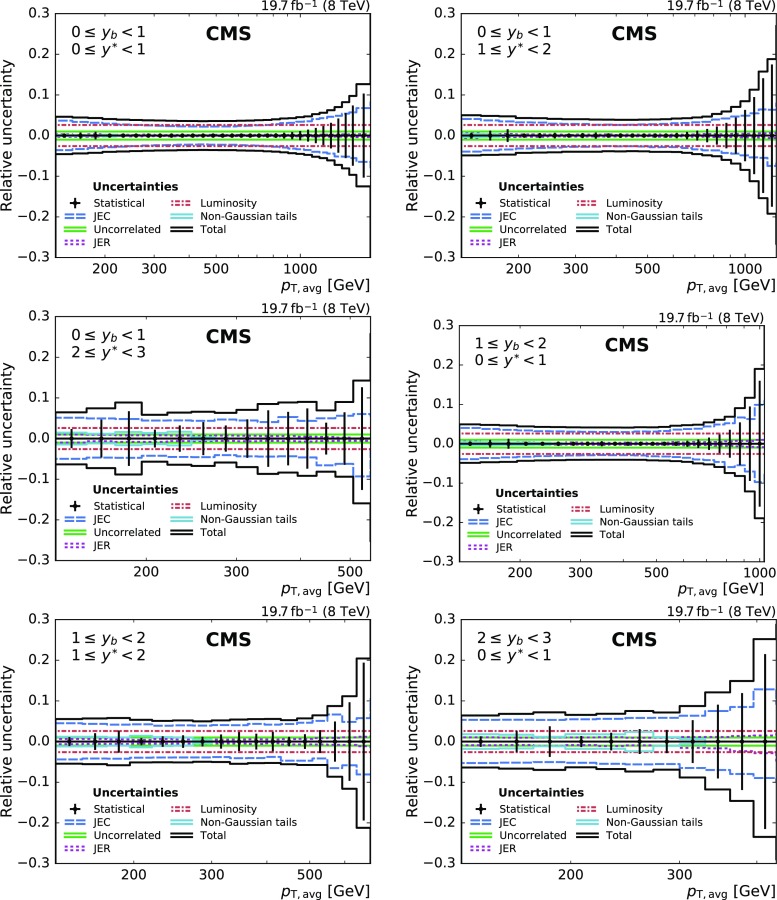
Overview of all experimental uncertainties affecting the cross section measurement in six bins of $y_{\mathrm {b}}$ and $y^{*}$. The error bars indicate the statistical uncertainty after unfolding. The different lines show the uncertainties resulting from jet energy corrections, jet energy resolution, integrated luminosity, non-Gaussian tails in the resolution, and from residual effects included in the uncorrelated uncertainty. The total uncertainty is obtained by adding all uncertainties in quadrature

The dominant systematic uncertainties in the cross section measurement arise from uncertainties in the JEC. Summing up quadratically all JEC uncertainties according to the prescription given in Ref. [23], the total JEC uncertainty amounts to about 2.5% in the central region and increases to 12% in the forward regions. The 2.6% uncertainty in the integrated luminosity [32] is directly propagated to the cross section. The uncertainty in the JER enters the measurement through the unfolding procedure and results in an additional uncertainty of 1–2% of the unfolded cross section. Non-Gaussian tails in the detector response to jets near $|y| = 3.0$, the maximal absolute rapidity considered in this measurement, are responsible for an additional uncertainty of up to 2%. Residual effects of small inefficiencies in the jet identification and trigger selection are covered by an uncorrelated uncertainty of 1% [11]. The total systematic experimental uncertainty ranges from about 3–8% in the central detector region and up to 12% for absolute rapidities near the selection limit of 3.0. Figure 3 depicts all experimental uncertainties as well as the total uncertainty, which is calculated as the quadratic sum of all the contributions from the individual sources.

## Theoretical predictions

**Fig. 4:**
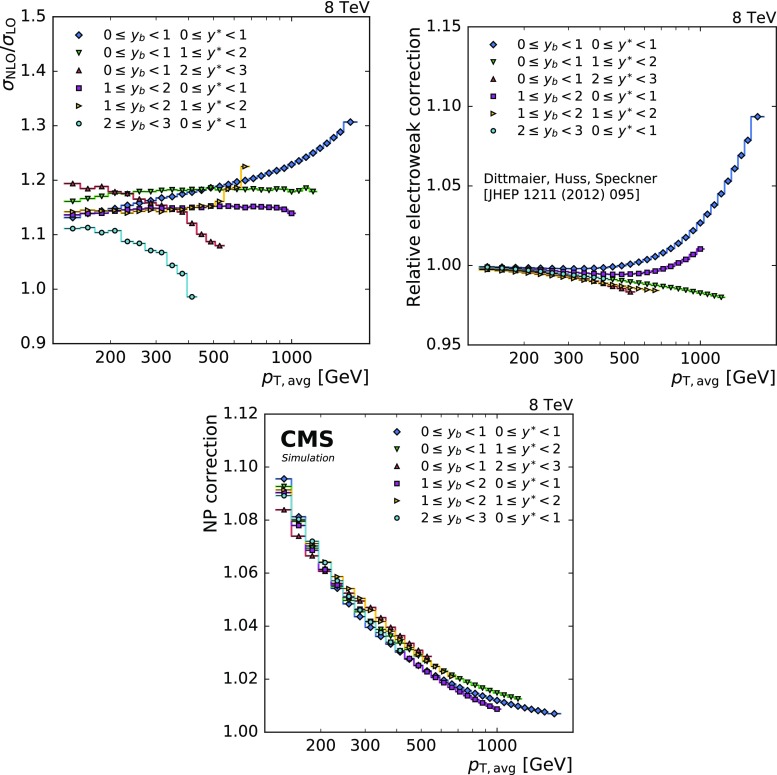
Overview of the theoretical correction factors. For each of the six analysis bins the NLO QCD (top left), the electroweak (top right), and the NP correction factor (bottom) are shown as a function of $p_{\mathrm {T,avg}}$. The NLO QCD correction has been derived with the same NLO PDF in numerator and denominator and is included in the NLO prediction by NLOJet++

The NLO predictions for the triple-differential dijet cross section are calculated using NLOJet++ within the framework of fastNLO version 2.1 [33, 34]. The renormalisation and factorisation scales $\mu _\text {r}$ and $\mu _\text {f}$ are both set to $\mu =\mu _0 =p_{\mathrm {T,max}} \cdot e^{0.3 y^{*}}$, a scale choice first investigated in Ref. [35]. The variation of these scales by constant factors as described below is conventionally used to estimate the effect of missing higher orders. The scale uncertainty is reduced in regions with large values of $y_{\mathrm {b}}$ with the above-mentioned choice for $\mu _0$ compared to a prediction with $\mu _0 =p_{\mathrm {T,avg}}$. The predictions for cross sections obtained with different central scale choices are compatible within the scale uncertainties. The calculation is performed using the PDF sets CT14, ABM11 [36], MMHT2014 [37], and NNPDF 3.0 [38] at next-to-leading evolution order which are accessed via the LHAPDF  6.1.6 interface [39, 40] using the respective values of $\alpha _S (M_\mathrm {Z})$ and the supplied $\alpha _S$ evolution. The size of the NLO correction is shown in Fig. 4 top left and varies between $+10$% and $+30$% at high $p_{\mathrm {T,avg}}$ and low $y_{\mathrm {b}}$.

The fixed-order calculations are accompanied by NP corrections, $c_k^\mathrm {NP}$, derived from the LO MC event generators pythia  8.185 [41] and herwig++  2.7.0 [42] with the tunes CUETP8M1 [43] and UE-EE-5C [44], respectively, and the NLO MC generator powheg  [45–48] in combination with pythia 8 and the tunes CUETP8M1 and CUETP8S1 [43].

The correction factor $c_{k}^{\mathrm {NP}}$ is defined as the ratio between the nominal cross section with and without multiple parton interactions (MPI) and hadronisation (HAD) effects

$$\begin{aligned} c_{k}^{\mathrm {NP}} = \frac{\sigma _{k}^{\mathrm {PS+HAD+MPI}}}{\sigma _{k}^{\mathrm {PS}}}\,, \end{aligned}$$

where the superscript indicates the steps in the simulation: the parton shower (PS), the MPI, and the hadronisation. The corresponding correction factor, as displayed in Fig. 4 bottom, is applied in each bin *k* to the parton-level NLO cross section. It differs from unity by about $+10$% for lowest $p_{\mathrm {T,avg}}$ and becomes negligible above 1$\,\text {TeV}$.

To account for differences among the correction factors obtained by using herwig++, pythia 8, and powheg+pythia 8, half of the envelope of all these predictions is taken as the uncertainty and the centre of the envelope is used as the central correction factor.

The contribution from EW effects, which arise mainly from virtual exchanges of massive W and Z bosons, is relevant at high jet $p_{\mathrm {T}}$ and central rapidities [49, 50]. These corrections, shown in Fig. 4 top right, are smaller than 3% below 1$\,\text {TeV}$ and reach 8% for the highest $p_{\mathrm {T,avg}}$. Theoretical uncertainties in this correction due to its renormalisation scheme and indirect PDF dependence are considered to be negligible.

**Fig. 5:**
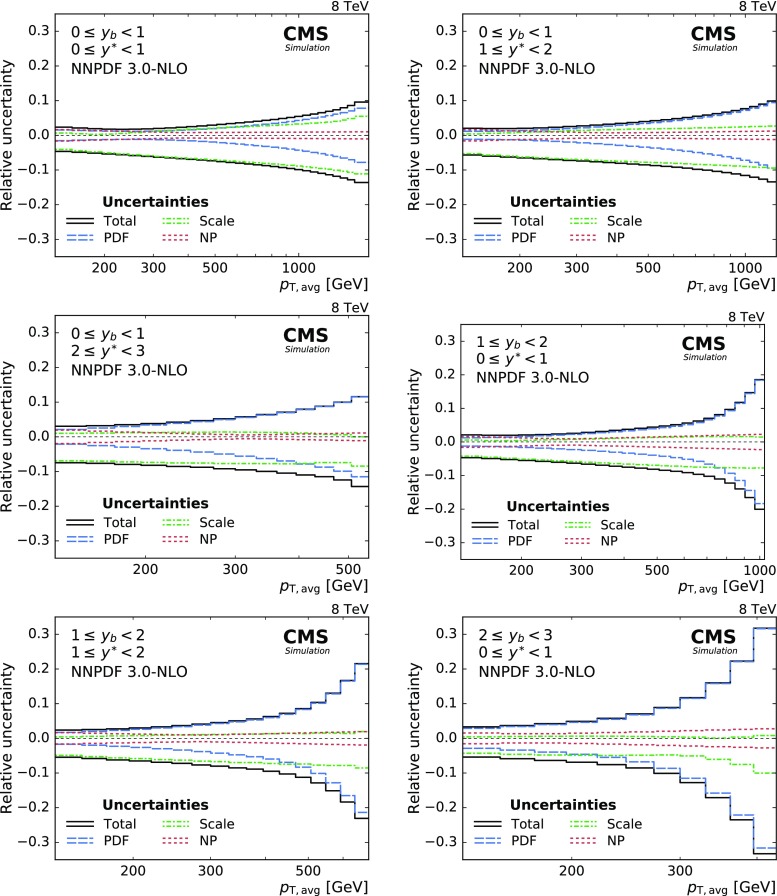
Overview of the theoretical uncertainties. The scale uncertainty dominates in the low-$p_{\mathrm {T,avg}}$ region. At high $p_{\mathrm {T,avg}}$, and especially in the boosted region, the PDFs become the dominant source of uncertainty

The total theoretical uncertainty is obtained as the quadratic sum of NP, scale, and PDF uncertainties. The scale uncertainties are calculated by varying $\mu _\text {r}$ and $\mu _\text {f}$ using multiplicative factors in the following six combinations: $(\mu _\text {r}/\mu _0, \mu _\text {f}/\mu _0) = (1/2, 1/2)$, (1 / 2, 1), (1, 1 / 2), (1, 2), (2, 1), and (2, 2). The uncertainty is determined as the maximal upwards and downwards variation with respect to the cross section obtained with the nominal scale setting [51, 52]. The PDF uncertainties are evaluated according to the NNPDF 3.0 prescription as the standard deviation from the average prediction. Figure 5 shows the relative size of the theoretical uncertainties for the phase-space regions studied. The scale uncertainty dominates in the low-$p_{\mathrm {T,avg}}$ region. At high $p_{\mathrm {T,avg}}$, and especially in the boosted region, the PDFs become the dominant source of uncertainty. In total, the theoretical uncertainty increases from about 2% at low $p_{\mathrm {T,avg}}$ to at least 10% and up to more than 30% for the highest accessed transverse momenta and rapidities.

## Results

**Fig. 6:**
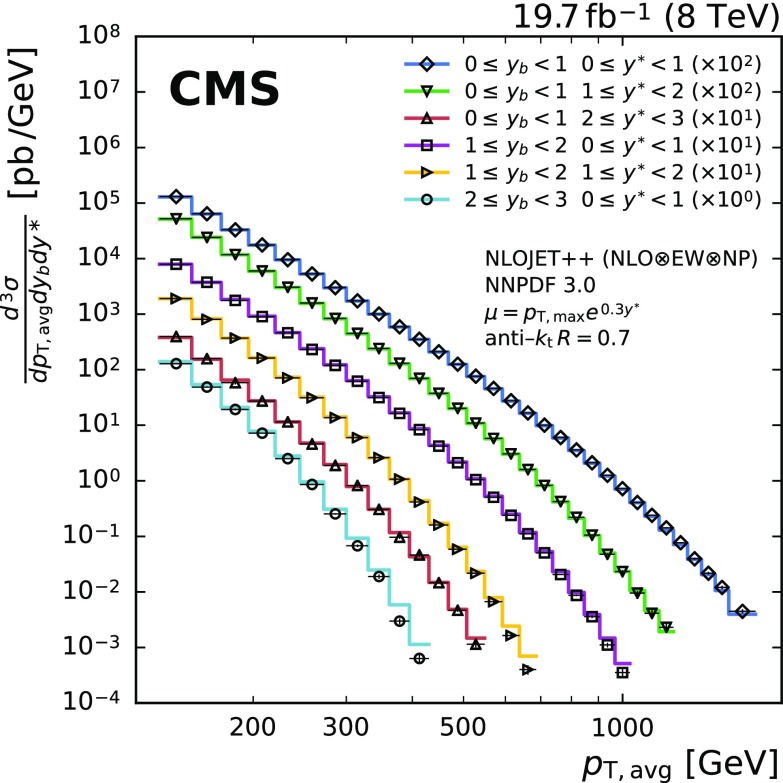
The triple-differential dijet cross section in six bins of $y^{*}$ and $y_{\mathrm {b}}$. The data are indicated by different markers for each bin. The theoretical predictions, obtained with NLOJet++ and NNPDF 3.0, and complemented with EW and NP corrections, are depicted by solid lines. Apart from the boosted region, the data are well described by the predictions at NLO accuracy over many orders of magnitude

The triple-differential dijet cross section is presented in Fig. 6 as a function of $p_{\mathrm {T,avg}}$ for six phase-space regions in $y^{*}$ and $y_{\mathrm {b}}$. The theoretical predictions are found to be compatible with the unfolded cross section over a wide range of the investigated phase space.

**Fig. 7:**
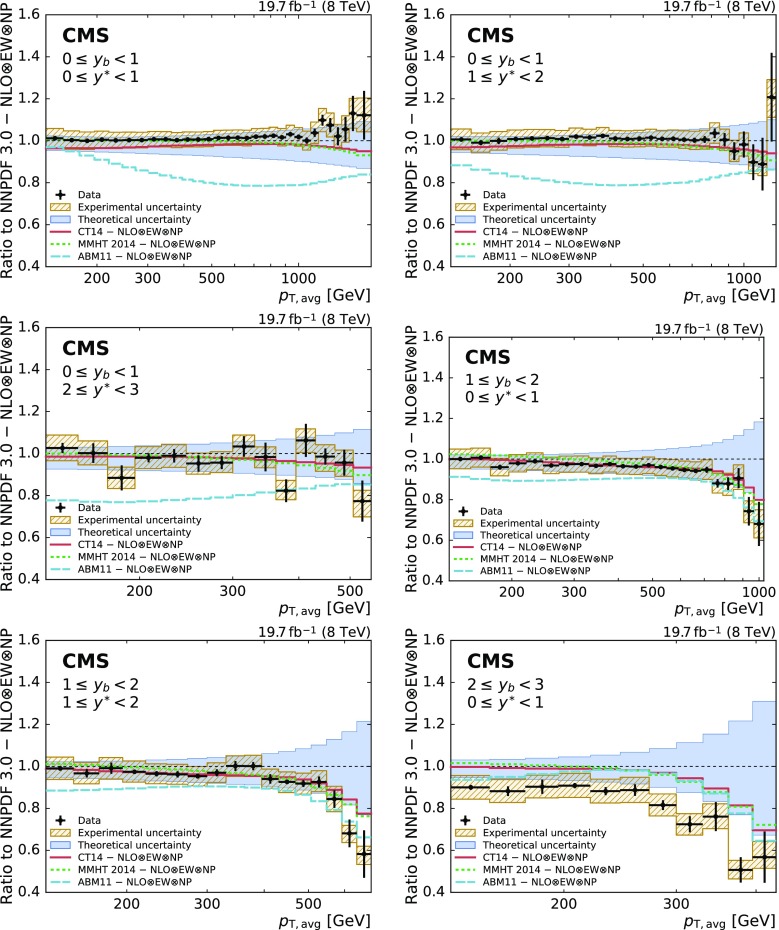
Ratio of the triple-differential dijet cross section to the NLOJet++ prediction using the NNPDF 3.0 set. The data points including statistical uncertainties are indicated by markers, the systematic experimental uncertainty is represented by the hatched band. The solid band shows the PDF, scale, and NP uncertainties quadratically added; the solid and dashed lines give the ratios calculated with the predictions for different PDF sets

The ratios of the measured cross section to the theoretical predictions from various global PDF sets are shown in Fig. 7. The data are well described by the predictions using the CT14, MMHT 2014, and NNPDF 3.0 PDF sets in most of the analysed phase space. In the boosted regions ($y_{\mathrm {b}} \ge 1$) differences between data and predictions are observed at high $p_{\mathrm {T,avg}}$, where the less known high-*x* region of the PDFs is probed. In this boosted dijet topology, the predictions exhibit large PDF uncertainties, as can be seen in Fig. 5. The significantly smaller uncertainties of the data in that region indicate their potential to constrain the PDFs.

Predictions using the ABM 11 PDFs systematically underestimate the data for $y_{\mathrm {b}} <2.0$. This behavior has been observed previously [53] and can be traced back to a soft gluon PDF accompanied with a low value of $\alpha _S (M_\mathrm {Z})$.

**Fig. 8:**
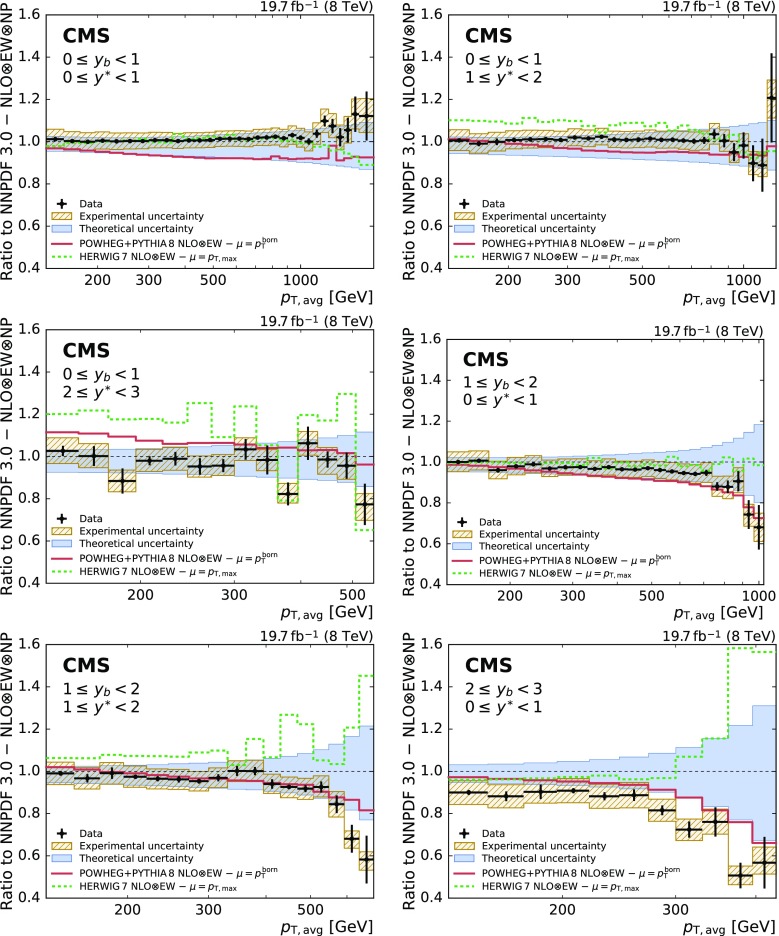
Ratio of the triple-differential dijet cross section to the NLOJet++ prediction using the NNPDF 3.0 set. The data points including statistical uncertainties are indicated by markers, the systematic experimental uncertainty is represented by the hatched band. The solid band shows the PDF, scale, and NP uncertainties quadratically added. The predictions of the NLO MC event generators powheg+pythia 8 and herwig 7 are depicted by solid and dashed lines, respectively

Figure 8 presents the ratios of the data to the predictions of the powheg+pythia 8 and herwig  7.0.3 [54] NLO MC event generators. Significant differences between the predictions from both MC event generators are observed. However, the scale definitions and the PDF sets are different. For powheg and herwig 7 the CT10 and MMHT 2014 PDF sets are used, respectively. In general, herwig 7 describes the data better in the central region whereas powheg prevails in the boosted region.

## PDF constraints and determination of the strong coupling constant

The constraints of the triple-differential dijet measurement on the proton PDFs are demonstrated by including the cross section in a PDF fit with inclusive measurements of deep-inelastic scattering (DIS) from the H1 and ZEUS experiments at the HERA collider [55]. The fit is performed with the open-source fitting framework xFitter version 1.2.2 [56]. The PDF evolution is based on the Dokshitzer–Gribov–Lipatov–Altarelli–Parisi (DGLAP) evolution equations [57–59] as implemented in the QCDNUM  17.01.12 package [60]. To ensure consistency between the HERA DIS and the dijet cross section calculations, the fits are performed at NLO.

The analysis is based on similar studies of inclusive jet data at 7$\,\text {TeV}$  [53] and 8$\,\text {TeV}$  [61] and all settings were chosen in accordance to the inclusive jet study at 8$\,\text {TeV}$  [61]. The parameterisation of the PDFs is defined at the starting scale $Q_0^2 = 1.9\,\text {GeV} ^2$. The five independent PDFs $xu_v(x)$, $xd_v(x)$, *xg*(*x*), $x\overline{U}(x)$, and $x\overline{D}(x)$ represent the u and d valence quarks, the gluon, and the up- and down-type sea quarks and are parameterised as follows:

1 $$\begin{aligned} xg(x)&= A_g x^{B_g} (1-x)^{C_g} - A_g' x^{B_g'}(1-x)^{C_g'}\,, \end{aligned}$$

2 $$\begin{aligned} xu_v(x)&= A_{u_{v}} x^{B_{u_{v}}} (1-x)^{C_{u_{v}}}(1 + D_{u_{v}}x+E_{u_{v}}x^2)\,,\end{aligned}$$

3 $$\begin{aligned} xd_v(x)&= A_{d_v} x^{B_{d_v}} (1-x)^{C_{d_{v}}}(1 + D_{d_{v}}x)\,,\end{aligned}$$

4 $$\begin{aligned} x\overline{U}(x)&= A_{\overline{U}} x^{B_{\overline{U}}} (1-x)^{C_{\overline{U}}}(1 + D_{\overline{U}}x)\,,\end{aligned}$$

5 $$\begin{aligned} x\overline{D}(x)&= A_{\overline{D}} x^{B_{\overline{D}}} (1-x)^{C_{\overline{D}}}\,, \end{aligned}$$

where $$x{\overline{U}}(x) = x{\overline{u}}(x)$$, and $$x{\overline{D}}(x) = x{\overline{d}}(x) + x{\overline{s}}(x)$$.

In these equations, the normalisation parameters $A_g$, $A_{u_{v}}$, and $A_{d_{v}}$ are fixed using QCD sum rules. The constraints $B_{\overline{U}}=B_{\overline{D}}$ and $A_{\overline{U}} = A_{\overline{D}}(1-f_s)$ are imposed to ensure the same normalisation for the $\overline{U}$ and $\overline{D}$ PDF for the $x \rightarrow 0$ region. The strange quark PDF is defined to be a fixed fraction $f_s = 0.31$ of $x\overline{D}(x)$. The generalised-mass variable-flavour number scheme as described in [62, 63] is used and the strong coupling constant is set to $\alpha _S (M_\mathrm {Z}) = 0.1180$. The set of parameters in Eqs. (1)–(5) is chosen by first performing a fit where all *D* and *E* parameters are set to zero. Further parameters are included into this set one at a time. The improvement of $\chi ^2$ of the fit is monitored and the procedure is stopped when no further improvement is observed. This leads to a 16-parameter fit. Due to differences in the sensitivity of the various PDFs to dijet and inclusive jet data, the parameterisation of the present analysis differs from that in Ref. [61]. In particular, the constraint $B_{d_v}=B_{u_v}$ at the starting scale has been released. This results in a d valence quark distribution consistent with the results obtained in Ref. [61] and in a similar CMS analysis of muon charge asymmetry in W boson production at 8$\,\text {TeV}$  [64].

The PDF uncertainties are determined using the HERAPDF method [55, 56] with uncertainties subdivided into the three categories of experimental, model, and parameterisation uncertainty, which are evaluated separately and added in quadrature to obtain the total PDF uncertainty.

Experimental uncertainties originate from statistical and systematic uncertainties in the data and are propagated to the PDFs using the Hessian eigenvector method [65] and a tolerance criterion of $\varDelta \chi ^2 = + 1$. Alternatively, the Monte Carlo method [66] is used to determine the PDF fit uncertainties and similar results are obtained.

The uncertainties in several input parameters in the PDF fits are combined into one model uncertainty. For the evaluation of the model uncertainties some variations on the input parameters are considered. The strangeness fraction is chosen in agreement with Ref. [67] to be $f_s=0.31$ and is varied between 0.23 and 0.39. Following Ref. [55], the b quark mass, set to $4.5 \,\text {GeV}$, is varied between 4.25 and $4.75\,\text {GeV}$. Similarly, the c quark mass, set by default to $1.47\,\text {GeV}$, is varied between 1.41 and $1.53\,\text {GeV}$. The minimum $Q^2$ imposed on the HERA DIS data is set in accordance with the CMS inclusive jet analysis described in [53] to $Q^2_\mathrm {min}=7.5\,\text {GeV} ^2$, and is varied between $Q^2_\mathrm {min} = 5.0\,\text {GeV} ^2$ and $10.0\,\text {GeV} ^2$.

The parameterisation uncertainty is estimated by including additional parameters in the fit, leading to a more flexible functional form of the PDFs. Each parameter is successively added in the PDF fit, and the envelope of all changes to the central PDF fit result is taken as parameterisation uncertainty. The increased flexibility of the PDFs while estimating the parameterisation uncertainty may lead to the seemingly paradoxical effect that, although new data are included, the total uncertainty can increase in regions, where direct constraints from data are absent. This may happen at very low or at very high *x*, where the PDF is determined through extrapolation alone. Furthermore, the variation of the starting scale $Q_0^2$ to 1.6 and 2.2$\,\text {GeV} ^2$ is considered in this parameterisation uncertainty.

The quality of the resulting PDF fit with and without the dijet measurement is reported in Table 2. The partial $\chi ^2$ per data point for each data set as well as the $\chi ^2/n_\text {dof}$ for all data sets demonstrate the compatibility of the CMS dijet measurement and the DIS data from the H1 and ZEUS experiments in a combined fit.

**Table 2:** The partial $\chi ^2$ ($\chi ^2_\text {p}$) for each data set in the HERA DIS (middle section) or the combined fit including the CMS triple-differential dijet data (right section) are shown. The bottom two lines show the total $\chi ^2$ and $\chi ^2/n_\text {dof}$. The difference between the sum of all $\chi ^2_\text {p}$ and the total $\chi ^2$ for the combined fit is attributed to the nuisance parameters

Data set	$n_{\mathrm {data}}$	HERA data		HERA & CMS data	
		$\chi ^2_\text {p}$	$\chi ^2_\text {p}/n_\text {data}$	$\chi ^2_\text {p}$	$\chi ^2_\text {p}/n_\text {data}$
NC HERA-I+II $\text {e}^{+}\text {p}$ $$E_{\mathrm {p}} = 920\,\text {GeV}\ $$	332	382.44	1.15	406.45	1.22
NC HERA-I+II $\text {e}^{+}\text {p}$ $E_{\mathrm {p}} = 820\,\text {GeV}$	63	60.62	0.96	61.01	0.97
NC HERA-I+II $\text {e}^{+}\text {p}$ $$E_{\mathrm {p}} = 575\,\text {GeV}\ $$	234	196.40	0.84	197.56	0.84
NC HERA-I+II $\text {e}^{+}\text {p}$ $$E_{\mathrm {p}} = 460\,\text {GeV}\ $$	187	204.42	1.09	205.50	1.10
NC HERA-I+II $\text {e}^{-}\text {p}$	159	217.27	1.37	219.17	1.38
CC HERA-I+II $\text {e}^{+}\text {p}$	39	43.26	1.11	42.29	1.08
CC HERA-I+II $\text {e}^{-}\text {p}$	42	49.11	1.17	55.35	1.32
CMS triple-differential dijet	122	–	–	111.13	0.91
Data set(s)	$n_{\mathrm {dof}}$	$\chi ^2$	$\chi ^2/n_\text {dof}$	$\chi ^2$	$\chi ^2/n_\text {dof}$
HERA data	1040	1211.00	1.16	–	–
HERA and CMS data	1162	–	–	1372.52	1.18

**Fig. 9:**
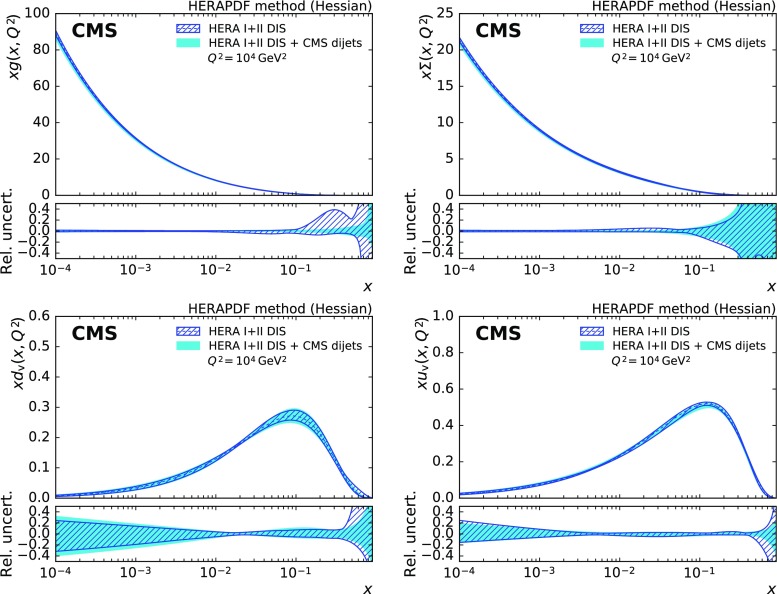
The gluon (top left), sea quark (top right), d valence quark (bottom left), and u valence quark (bottom right) PDFs as a function of *x* as derived from HERA inclusive DIS data alone (hatched band) and in combination with CMS dijet data (solid band). The PDFs are shown at the scale $Q^2 = 10^{4}\,\text {GeV} ^2$ with their total uncertainties

The PDFs obtained for the gluon, u valence, d valence, and sea quarks are presented for a fit with and without the CMS dijet data in Fig. 9 for $Q^2=10^4\,\text {GeV} ^2$. The uncertainty in the gluon PDF is reduced over a large range in *x* with the largest impact in the high-*x* region, where some reduction in uncertainty can also be observed for the valence quark and the sea quark PDFs. For *x* values beyond ${\approx }\, 0.7$ or below $10^{-3}$, the extracted PDFs are not directly constrained by data and should be considered as extrapolations that rely on PDF parameterisation assumptions alone.

**Fig. 10:**
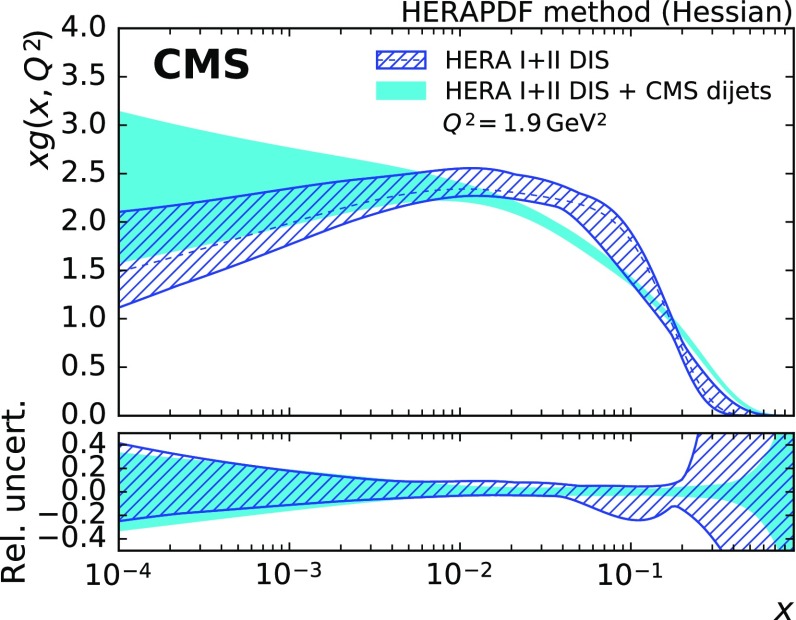
The gluon PDF as a function of *x* as derived from HERA inclusive DIS data alone (hatched band) and in combination with CMS dijet data (solid band). The PDF and its total uncertainty are shown at the starting scale $Q^2 = 1.9\,\text {GeV} ^2$ of the PDF evolution

The improvement in the uncertainty of the gluon PDF is accompanied by a noticeable change in shape, which is most visible when evolved to low scales as shown in Fig. 10. Compared to the fit with HERA DIS data alone, the gluon PDF shrinks at medium *x* and increases at high *x*. A similar effect has been observed before, e.g. in Ref. [53].

**Fig. 11:**
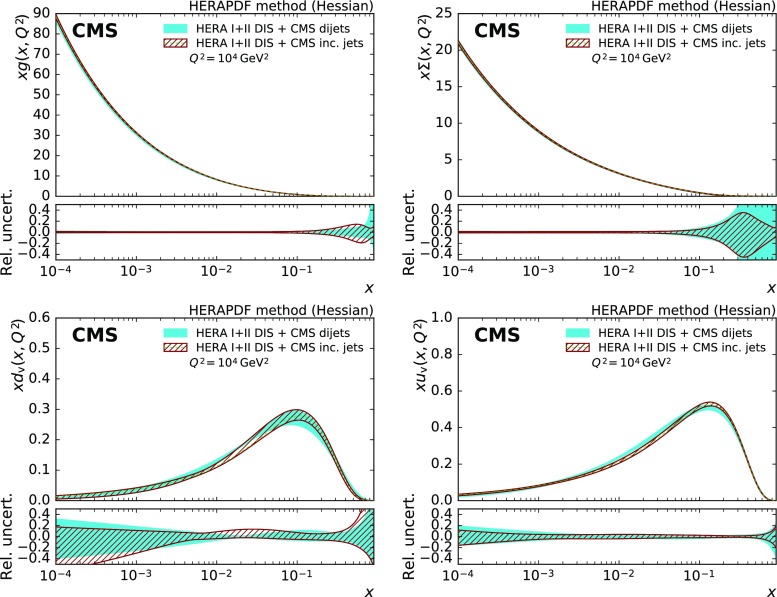
The gluon (top left), sea quark (top right), d valence quark (bottom left), and u valence quark (bottom right) PDFs as a function of *x* as derived from a fit of HERA inclusive DIS data in combination with CMS inclusive jet data (solid band) and CMS dijet data (hatched band) at 8 TeV. The PDFs are shown at the scale $Q^2 = 10^{4}\,\text {GeV} ^2$ with their total uncertainties

The PDFs are compared in Fig. 11 to those obtained with inclusive jet data at $\sqrt{s} = 8\,\text {TeV}$ [61]. The shapes of the PDFs and the uncertainties are similar. Somewhat larger uncertainties in the valence quark distributions are observed in the fit using the dijet data with respect to those obtained from the inclusive jet cross section. This behaviour can be explained by a stronger sensitivity of the dijet data to the light quark distributions, resulting in an increased flexibility of the PDF parameterisation, however, at the cost of an increased uncertainty.

The measurement of the triple-differential dijet cross section not only provides constraints on the PDFs, but also on the strong coupling constant. Therefore, the PDF fit is repeated with an additional free parameter: the strong coupling constant $\alpha _S (M_\mathrm {Z})$. The value obtained for the strong coupling constant is

$$\begin{aligned} \alpha _S (M_\mathrm {Z}) = 0.1199\,\pm \,0.0015(\mathrm {exp})_{-0.0002}^{+0.0002}(\mathrm {mod})_{-0.0004}^{+0.0002}(\mathrm {par}), \end{aligned}$$

where the quoted experimental (exp) uncertainty accounts for all sources of uncertainties in the HERA and CMS data sets, as well as the NP uncertainties. The model (mod) and parameterisation (par) uncertainties are evaluated in the same way as in the PDF determination. The consideration of scale uncertainties in a global PDF fit is an open issue in the PDF community because it is unclear how to deal with the correlations in scale settings among the different measurements and observables. Therefore they are not taken into account in any global PDF fit up to now, although an elaborate study of the effect of scale settings on dijet cross sections has been performed in Ref. [68], which also reports first combined PDF and $\alpha _S (M_\mathrm {Z})$ fits using LHC inclusive jet data. Following Ref. [53], where the final uncertainties and correlations of CMS inclusive jet data at 7$\,\text {TeV}$ are used in such combined fits, two different methods to evaluate the scale uncertainty of the jet cross section on $\alpha _S (M_\mathrm {Z})$ are studied. First, the renormalisation and factorisation scales are varied in the calculation of the dijet predictions. The fit is repeated for each variation. The uncertainty is evaluated as detailed in Sect. 5 and yields $\varDelta \alpha _S (M_\mathrm {Z}) = ^{+0.0026}_{-0.0016}(\text {scale, refit})$.

The second procedure is analogous to the method applied by CMS in previous determinations of $\alpha _S (M_\mathrm {Z})$ without simultaneous PDF fits, cf. Refs. [53, 61, 69, 70]. The PDFs are derived for a series of fixed values of $\alpha _S (M_\mathrm {Z})$ and the nominal choice of $\mu _\text {r}$ and $\mu _\text {f}$. Using this series, the best fit $\alpha _S (M_\mathrm {Z})$ value of the dijet data is determined for each scale variation. Here, the evaluated uncertainty is $\varDelta \alpha _S (M_\mathrm {Z}) = ^{+0.0031}_{-0.0019}(\text {scale}, \alpha _S (M_\mathrm {Z}) \text {series})$.

Both results, $\alpha _S (M_\mathrm {Z}) = 0.1199 ^{+0.0015}_{-0.0016}$ (all except scale) with $^{+0.0026}_{-0.0016}$ (scale, refit) and $^{+0.0031}_{-0.0019}$ (scale, $\alpha _S (M_\mathrm {Z})$ series), are in agreement with Ref. [53], which reports $\alpha _S (M_\mathrm {Z}) = 0.1192 ^{+0.0023}_{-0.0019}$ (all except scale) and $^{+0.0022}_{-0.0009}$ (scale, refit) respectively $^{+0.0024}_{-0.0039}$ (scale, $\alpha _S (M_\mathrm {Z})$ series). Similarly, it is observed that the second procedure leads to somewhat larger scale uncertainties, because there is less freedom for compensating effects between different gluon distributions and the $\alpha _S (M_\mathrm {Z})$ values. Since this latter uncertainty is the most consistent to be compared with previous fixed-PDF determinations of $\alpha _S (M_\mathrm {Z})$, it is quoted as the main result. The dominant source of uncertainty is of theoretical origin and arises due to missing higher order corrections, whose effect is estimated by scale variations.

This value of $\alpha _S (M_\mathrm {Z})$ is in agreement with the results from other measurements by CMS [53, 61, 69–71] and ATLAS [72], with the value obtained in a similar analysis complementing the DIS data of the HERAPDF2.0 fit with HERA jet data [55], and with the world average of $\alpha _S (M_\mathrm {Z}) =0.1181\pm 0.0011$ [73]. In contrast to the other CMS results, this analysis is mainly focused on PDF constraints. The running of the strong coupling constant was tested only indirectly via the renormalisation group equations. No explicit test of the running was carried out by subdividing the phase space into regions corresponding to different values of the renormalisation scale.

## Summary

A measurement of the triple-differential dijet cross section is presented for $\sqrt{s}=8\,\text {TeV}$. The data are found to be well described by NLO predictions corrected for nonperturbative and electroweak effects, except for highly boosted event topologies that suffer from large uncertainties in parton distribution functions (PDFs).

The precise data constrain the PDFs, especially in the highly boosted regime that probes the highest fractions *x* of the proton momentum carried by a parton. The impact of the data on the PDFs is demonstrated by performing a simultaneous fit to cross sections of deep-inelastic scattering obtained by the HERA experiments and the dijet cross section measured in this analysis. When including the dijet data, an increased gluon PDF at high *x* is obtained and the overall uncertainties of the PDFs, especially those of the gluon distribution, are significantly reduced. In contrast to a fit that uses inclusive jet data, this measurement carries more information on the valence-quark content of the proton such that a more flexible parameterisation is needed to describe the low-*x* behaviour of the u and d valence quark PDFs. This higher sensitivity is accompanied by slightly larger uncertainties in the valence quark distributions as a consequence of the greater flexibility in the parameterisation of the PDFs.

In a simultaneous fit the strong coupling constant $\alpha _S (M_\mathrm {Z})$ is extracted together with the PDFs. The value obtained at the mass of the Z boson is

$$\begin{aligned} \alpha _S (M_\mathrm {Z})&= 0.1199\, \pm {0.0015}\,(\mathrm {exp})\\&\quad \pm {0.0002}\,(\mathrm {mod}) \,{}_{-0.0004}^{+0.0002}\,(\mathrm {par})\, {}_{-0.0019}^{+0.0031}\,(\mathrm {scale})\\&= 0.1199\, \pm {0.0015}\,(\mathrm {exp})\, _{-0.0020}^{+0.0031}\,(\mathrm {theo}), \end{aligned}$$

and is in agreement with previous measurements at the LHC by CMS [53, 61, 69–71] and ATLAS [72], and with the world average value of $\alpha _S (M_\mathrm {Z}) = 0.1181 \,\pm \, 0.0011$ [73]. The dominant uncertainty is theoretical in nature and is expected to be reduced significantly in the future using pQCD predictions at next-to-next-to-leading order [74].
